# Trichostomatid Ciliates (Alveolata, Ciliophora, Trichostomatia) Systematics and Diversity: Past, Present, and Future

**DOI:** 10.3389/fmicb.2019.02967

**Published:** 2020-01-15

**Authors:** Franciane Cedrola, Marcus Vinicius Xavier Senra, Mariana Fonseca Rossi, Priscila Fregulia, Marta D’Agosto, Roberto Júnio Pedroso Dias

**Affiliations:** ^1^Laboratório de Protozoologia, Programa de Pós-graduação em Comportamento e Biologia Animal, Instituto de Ciências Biológicas, Universidade Federal de Juiz de Fora, Juiz de Fora, Brazil; ^2^Instituto de Recursos Naturais Renováveis, Universidade Federal de Itajubá, Itajubá, Brazil

**Keywords:** Entodiniomorphida, integrative taxonomy, Macropodiniida, symbiotic ciliates, Vestibuliferida

## Abstract

The gastrointestinal tracts of most herbivorous mammals are colonized by symbiotic ciliates of the subclass Trichostomatia, which form a well-supported monophyletic group, currently composed by ∼1,000 species, 129 genera, and 21 families, distributed into three orders, Entodiniomorphida, Macropodiniida, and Vestibuliferida. In recent years, trichostomatid ciliates have been playing a part in many relevant functional studies, such as those focusing in host feeding efficiency optimization and those investigating their role in the gastrointestinal methanogenesis, as many trichostomatids are known to establish endosymbiotic associations with methanogenic Archaea. However, the systematics of trichostomatids presents many inconsistencies. Here, we stress the importance of more taxonomic works, to improve classification schemes of this group of organisms, preparing the ground to proper development of such relevant applied works. We will present a historical review of the systematics of the subclass Trichostomatia highlighting taxonomic problems and inconsistencies. Further on, we will discuss possible solutions to these issues and propose future directions to leverage our comprehension about taxonomy and evolution of these symbiotic microeukaryotes.

## Introduction

The gastrointestinal tracts of most herbivorous mammals are colonized by symbiotic ciliates of the subclass Trichostomatia Bütschli, 1889 (Supplementary Video S1). These play a central role for the efficient fermentative process in the host intestinal tract and also contribute to the degradation process of proteins, lipids, nitrogen compounds and carbohydrates, such as cellulose, hemicellulose and starch (Dehority, 1986; Wright, 2015). These microeukaryotes form a well-supported monophyletic group, currently composed of ∼1,000 species, 129 genera, and 21 families (Supplementary  Material S1) that are distributed across three orders: Entodiniomorphida Reichenow, in Doflein & Reichenow, 1929, including species with ciliary zones restricted to tufts or bands, and infraciliatures organized as polybrachykineties, Macropodiniida Lynn, 2008 and Vestibuliferida de Puytorac et al., 1974, including ciliates all covered by cilia and with a densely ciliated vestibulum (Lynn, 2008; Cedrola et al., 2015; Gao et al., 2016). In recent years, trichostomatid ciliates have been playing a part in many relevant functional studies, such as those focusing on host feed efficiency optimization (Newbold et al., 2015) and those investigating their role in gastrointestinal methanogenesis, as many trichostomatids are known to establish endosymbiotic associations with methanogenic Archaea (Embley et al., 2003). Methanogenesis from ciliate associated methanogens may account for up to 60% of methane emissions into the Earth’s atmosphere (Intergovernmental Panel on Climate Change [IPCC], 2019; Malmuthuge and Guan, 2017). However, the systematics of trichostomatids presents many inconsistencies. Here, we stress the importance of more taxonomic works, to improve classification schemes of this group of microorganisms. This will provide a sound basis for ciliate community structure assessment. We present a historical review of the systematics of the subclass Trichostomatia highlighting taxonomic problems and inconsistencies. We also discuss possible solutions and propose future directions to broaden our understanding of the taxonomy and evolution of these symbiotic microeukaryotes.

## Past

Trichostomatid ciliates were discovered in the first half of the 19th century by Gruby and Delafond (1843). However, the authors, presented only a brief and succinct report about high densities of “animaculous” inhabiting the stomach and intestine of domestic cattle and horses. The first illustrations of trichostomatid ciliates are attributed to Colin (1854) while the author studied domestic mammals. G. Colin performed live observations of many species, possibly including members of the genera *Blepharocorys* Bundle, 1895, *Bundleia* da Cunha and Muniz, 1928, *Cycloposthium* Bundle, 1896, *Diplodinium* Schuberg, 1888 and *Entodinium* Stein, 1859. The first author to publish a formal taxonomic work on trichostomatid ciliates was F. Stein (1858) describing, although superficially, species of the genera *Entodinium*, *Isotricha*, and *Ophryoscolex* and the family Ophryoscolecidae. Following, several novel species were described from many geographic locations and from different host species. In this period, beginning with the work of F. Stein (1858) until the late 1970s, more than 400 species were described, indicating that trichostomatid ciliates may constitute a diverse group of microorganisms (Fiorentini, 1889; Bundle, 1895; Poche, 1913; Da Cunha, 1914a,b; Gassovsky, 1919; Buisson, 1923a,b,c, 1924; Crawley, 1923; Dogiel,1925a,b, 1926a,b, 1927, 1928, 1932, 1934, 1935; Fantham, 1926; Becker and Talbot, 1927; Hsiung, 1930, 1935a,b, 1936; Kofoid and MacLennan, 1930, 1932, 1933; Jirovec, 1933; Kofoid and Christenson, 1933; Kofoid, 1935; Wertheim, 1935; Fonseca, 1939; Moriggi, 1941; Sládeček, 1946; Bush and Kofoid, 1948; Lubinsky, 1957a,1958a,b; Latteur, 1966a,b, 1967, 1968, 1969, 1970; Wolska, 1967b, 1968, 1969). Most of these studies were done based only on live observations and by using simple ciliatological techniques, such as hematoxylin and iodine staining methods, which were the available tools at that time. Nevertheless, many morphological characters, such as skeletal plates (Dogiel, 1923; Schulze, 1924, 1927; Dogiel and Fedorowa, 1925), contractile vacuoles (Kraschnninikow, 1929; MacLennan, 1933), concretion vacuoles (Dogiel, 1929), and paralabial organelles (Bretschneider, 1962) could be clearly characterized, allowing the inclusion of these microeukaryotes into the phylum Ciliophora, orders Entodiniomorphida and Vestibuliferida (for history of classification, see Supplementary Material S2). In this same period, the first studies appeared that proposed hypotheses on the evolution of this group of microorganisms. According to Dogiel (1947) and Lubinsky (1957a, b, c), within the family Ophryoscolecidae, subfamily Entodiniinae could be considered ancestral due to its characteristic single ciliary zone, single contractile vacuole, poorly developed caudal spines and lack of skeletal plates. The Ophryoscolecinae is considered to be the most recent group for presenting two ciliary zones, large number of vacuoles and skeletal plates, and developed caudal projections. Diplodiniinae is considered an intermediate group.

The development of silver impregnation techniques in 1930s (Bodian, 1936, 1937), which can reveal in details infraciliary and other argentophilic structures patterns, represented a great revolution in the systematics of Ciliophora (Lynn, 2008). They were initially applied to trichostomatids by Noirot-Timothée (1956a, b) where the infraciliary band patterns of *Epidinium* Crawley, 1923 and *Ophryoscolex* Stein, 1858 were described. Further studies were performed by several authors and contributed to our understanding of infraciliary band patterns in various trichostomatid ciliate species (Noirot-Timothée, 1960; Grain, 1962, 1963a,b, 1964, 1965; Batisse, 1966). However, the greatest contribution was achieved by M. Wolska in a series of seminal works (Wolska, 1963, 1964, 1965, 1966a,b, 1967a,b, 1968, 1969, 1970, 1971a,b, 1978a,b,c,d, 1979, 1985, 1986), which described infraciliary band patterns and morphogenetic processes in ciliates of the families Buetschliidae Poche, 1913, Blepharocorythidae Hsiung, 1929, Spirodiniidae Strelkow, 1939, Pseudoentodiniidae Wolska, 1985 (Entodiniomorphida), Isotrichidae Bütschli, 1889 and Paraisotrichidae Da Cunha, 1915 (Vestibuliferida). As a result of these detailed investigations, a hypothesis on the evolutionary relationship within the Trichostomatia was proposed by Wolska (1971b). According to the descriptions there are several patterns of infraciliary bands in Trichostomatia in which are composed by at least one of these bands: adoral polybrachykinety, dorsal polybrachykinety, dorso-adoral polybrachykinety, kinety loop, paralabial kineties, vestibular polybrachykinety, and vestibular kineties (Supplementary Figure S1).

Ultrastructural works also impacted the systematics of trichostomatid ciliates. Bonhomme (1989), after collecting data on the ultrastructure of many Entodiniomorphina (order Entodiniomorphida) representatives, suggested that this suborder could be classified into two groups, according to their cortex ultrastructure information. The first is composed of ciliates with the cortex lacking dense longitudinal cords (genus *Cycloposthium* Bundle, 1895; Ophryoscolecidae Stein, 1859 and Troglodytellidae Corliss, 1979), and the second is composed of ciliates with dense longitudinal cords (genus *Tripalmaria* and Spirodiniidae Strelkow, 1939).

Further, based on a compilation of structural and ultrastructural data, Small and Lynn (1981) proposed Trichostomatia as a subclass of the class Litostomatea, and as a sister group of the subclass Haptoria Corliss, 1974.

Over the last 30 years, after a long period of scarce taxonomic data being produced, many taxonomic inventories of trichostomatids isolated from several mammalian host species, domestic and wild, from different geographic locations (Supplementary Table S1) started to appear in the literature, leading to the characterization of a series of novel species, including trichostomatids inhabiting the gastrointestinal tracts of Australian marsupials (Dehority, 1996; Cameron et al., 2000a,b, 2001a,b, 2002, 2003; Cameron and O’Donoghue, 2001, 2002a,b, 2002c,2003a,b,c, 2004a,b). These ciliates present several exclusive morphological features among trichostomatids. For this reason, Lynn (2008) proposed the creation of a new order to include them, Macropodiniida. This period was also characterized by the establishment of new silver impregnation techniques for trichostomatid ciliates, such as the adaptations of ammoniacal silver carbonate impregnation proposed by Ito and Imai (1998) and Rossi et al. (2016) and the adaption of Protargol’s impregnation for vestibuliferids proposed by Ito and Imai (2000). These techniques allowed the development of several studies describing the infraciliature and morphogenetic process in different trichostomatid species (Ito et al., 1997, 2001, 2002, 2006, 2008, 2010, 2011, 2014, 2017, 2018; Ito and Imai, 1998, 2003, 2005, 2006; Gürelli and Ito, 2014; Cedrola et al., 2016, Cedrola et al., 2017a, b, 2018a,b; Gürelli and Akman, 2016; Gürelli, 2018, 2019; Ito and Tokiwa, 2018), which were very important to understand the evolutionary relationships within the Trichostomatia.

A novel view on the systematics of trichostomatid ciliates emerged in the late 1990s with the advent of molecular techniques. The first molecular phylogenies (Wright and Lynn, 1997a,b,c; Wright et al., 1997) corroborated the initial morphological studies placing trichostomatids as a monophyletic group within the Litostomatea. Starting from early 2000s and with the increasing availability of 18S rRNA gene sequences of members of the subclass Trichostomatia in public repositories (Cameron et al., 2001a, 2003; Cameron and O’Donoghue, 2004b; Strüder-Kypke et al., 2007; Ito et al., 2010, 2014; Pomajbíková et al., 2010, 2013; Snelling et al., 2011; Chistyakova et al., 2014; Moon-Van der Staay et al., 2014; Grim et al., 2015; Kittelmann et al., 2015; Rossi et al., 2015; Bardele et al., 2017; Cedrola et al., 2017, 2019), the internal phylogenetic relationships within the subclass began to be elucidated. This caused a revolution in their systematics and revealed several taxonomic incongruences, mainly with respect to Entodiniomorphida and Vestibuliferida, for which the grouping based on morphological features does not seem to hold.

## Present

**FIGURE 1:**
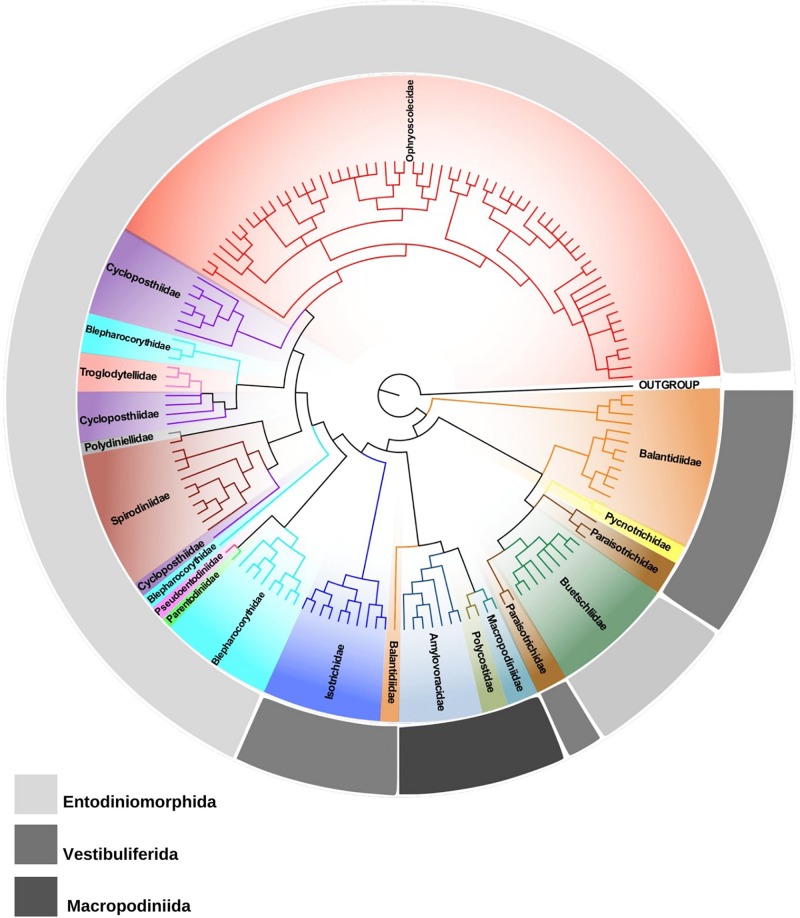
Phylogenetic tree of trichostomatid ciliates (Ciliophora, Litostomatea, and Trichostomatia) estimated by Bayesian Inference and based on 18S rRNA gene data. *Spathidium papilliferum* was chosen as out group.

Currently, the subclass Trichostomatia consists of three major orders, Entodiniomorphida, Macropodiniida, and Vestibuliferida. Macropodiniida is the only group for which multidisciplinary taxonomic approaches were applied (Cameron and O’Donoghue, 2001, 2002a,b,c, 2003a,b,c, 2004a,b; Cameron et al., 2000a,b,2001a,b, 2002, 2003). Their representatives are distributed in three monophyletic families all with well-supported internal nodes (Figure 1 and Supplementary Figure S2). However, most of the species diversity of Trichostomatia occurs within the Entodiniomorphida and Vestibuliferida, which are extremely neglected groups concerning taxonomic studies. According to 18S rRNA gene reconstructions (Figure 1 and Supplementary Figure S2; Ito et al., 2014; Kittelmann et al., 2015), the order Entodiniomorphida is not monophyletic, emerging in the tree as two independent clades, one containing representatives of the families Blepharocorythidae Hsiung, 1929, Parentodiniidae Ito et al., 2002, Pseudoentodiniidae Wolska, 1986, Cycloposthiidae Poche, 1913, Spirodiniidae Strelkow, 1939, Polydiniellidae Corliss, 1960, Troglodytellidae Corliss, 1979, and Ophrysocolecidae Stein, 1859; and another containing members of the family Buetschliidae Poche, 1913. Moreover, many of these families do not constitute natural groups, such as Blepharocorythidae, Cycloposthiidae, and Spirodiniidae; and for those that are monophyletic, such as Ophryoscolecidae, the internal branching is poorly supported, as detected in previous works (Ito et al., 2014; Kittelmann et al., 2015; Rossi et al., 2015; Cedrola et al., 2017). Many inconsistencies can also be observed in the order Vestibuliferida with representatives distributed in three distinct clades (Figure 1 and Supplementary Figure S2; Ito et al., 2014; Kittelmann et al., 2015), in which the families Balantididae and Paraisotrichidae do not constitute natural groups. Moreover, 18S rRNA gene sequences are only available from representatives of 16 out of the 21 currently recognized families of Trichostomatia. The families with no molecular data are: Gilchristinidae (Ito et al., 2014), Rhinozetidae Van Hoven et al., 1988, Telamonididae Latteur and Dufey, 1967 (Entodiniomorphida), Protocaviellidae Grain and Corliss, 1979, Protohallidae Cunha and Muniz, 1927 (Vestibuliferida). Still, many of the existing families of which molecular data are available, such as Polydiniellidae Corliss, 1960, Troglodytellidae Corliss, 1979 (Entodiniomorphida) and Pycnotrichidae Poche, 1913 (Vestibuliferida) have only one representative with its 18S rRNA gene sequenced, limiting the power of phylogenetic reconstructions within the whole group. The scarcity and absence of consistent morphological data from many trichostomatid groups is also of concerns, for example, there are no structural (infraciliary pattern and morphogenesis) and ultrastructural data described for many cycloposthiids, troglodytelids, and spirodinids, which makes it impossible to establish homology hypotheses on trichostomatids. Moreover, the lack of detailed morphological data contributes to taxonomic inconsistencies and hinders the development of novel classifications schemes that reflect evolutionary divergences.

## Future

Despite the great advances obtained after implementing silver staining, ultrastructural and molecular methods, it is clear that huge gaps are still preventing a cohesive systematic scheme of Trichostomatia, especially when we compare the existing data with other Ciliophora groups (Warren et al., 2017). In the forthcoming years, we need to invest more in detailed descriptions and redescriptions of infraciliary band patterns and morphogenesis, on 18S rRNA gene sequencing, and in depth ultrastructure characterizations. Using these methods, we need to study trichostomatids from a wide variety of hosts especially in so far neglected geographical regions such as, e.g., neotropical areas, with emphasis on Entodiniomorphida and Vestibuliferida. We should further expand this work to trichostomatid families such as the Protocaviellidae and Protohallidae from domestic and wild rodents and Gilchristinidae, Rhinozetidae, and Telamonididae from elephants, rhinos and wild pigs, respectively. Moreover, improvements to trichostomatid cultivation techniques, which are still poorly developed (Williams and Coleman, 1992; Dehority and Wright, 2014; Newbold et al., 2015; Belzecki et al., 2016), would be of great importance to obtain suitable samples for morphology and molecular characterization approaches. Collectively, this information will contribute to develop more robust phylogenetic hypotheses, to elaborate taxonomic reformulations, contributing to elucidate the many taxonomic incongruences presented above and to establish new classification schemes that reflect evolutionary divergences within Trichostomatia.

Apart from 18S rRNA genes, it is time to obtain data on other informative loci from pure/axenic cultures, such as the internal transcribed spacer region and 28S ribosomal RNA genes, to further improve our understanding of the phylogenetic relationships within the Litostomatea (Rajter and Vd’ačný, 2017). In addition, it is possible to identify new macronuclear regions, using genomic information of Trichostomatia representatives (Park et al., 2018), and to obtain hydrogenosomal sequences, such as those from 16S and Fe-Hydrogenase. Also, it is possible to use the next generation sequencing techniques to perform phylogenomic reconstruction, as done for other Ciliophora groups within the last decade (Feng et al., 2015; Gentekaki et al., 2017; Jiang et al., 2019). This data could be used in macro-evolutionary approaches to reveal divergence times and the mode of evolution in trichostomatid ciliates. The timescale and evolutionary dynamics of these symbiotic ciliates are yet to be determined (Newbold et al., 2015). Molecular dating studies are restricted to Wright and Lynn (1997c) and Vd’ačný (2015, 2018), which employed different molecular dating methods, taxon sampling and calibration data, using mostly the fossil record of hosts and the posterior ages estimated from previous studies as calibration priors for ciliates time tree. Baele et al. (2006) provided evidence for the presence of numerous heterotachous sites (sites in which its substitution rates can vary with time) within the 18S rRNA gene of ciliates, which may result in the introduction of bias. Thus, further improvements to the calculation and resolution of trichostomatid phylogenies are needed through the use of evolutionary models, such as, for example, the mixture of branch lengths (MBL) (Zhou et al., 2007).

## Data Availability Statement

The datasets generated for the phylogenetic analyses are available on request to the corresponding author.

## Author Contributions

FC, PF, and MD collected the data. FC, MS, and RD participated in the conception of the study. FC, MS, and MR participated in the manuscript writing. FC, MR, and PF prepared the figures and supplementary material. All authors have read and approved the final manuscript.

## Conflict of Interest

The authors declare that the research was conducted in the absence of any commercial or financial relationships that could be construed as a potential conflict of interest.

## Supplementary Material

FIGURE S1 Oral infraciliary bands pattern of Trichostomatid ciliates. **A–D**. Order Vestibuliferida. **A**, Isotrichidae; **B**, Paraisotrichidae; **C**, Protocaviellidae; **D**, Protohallidae; **E–L**, Order Entodiniomorphida; **E**, Buetschliidae; **F**, Blepharocorythidae; **G**, Cycloposthiidae; **H**, Gichristinidae; **I**, Ophryoscolecidae; **J**, Parentodiniidae; **K**, Pseudoentodiniidae; **L**, Spirodiniidae; **M–O**, Order Macropodiniida; **M**, Amylovoracidae; **N**, Polycostidae; **O**, Macropodiniidae; AP, adoral polybrachykinety; CB, cytopharyngeal basket; DAP, dorso-adoral polybrachykinety; PVP, perivestibular polybrachykinety, and VK, vestibular kineties.

FIGURE S2 Phylogenetic tree of trichostomatid ciliates (Ciliophora, Litostomatea, and Trichostomatia) based on 18S rRNA gene data. *Spathidium papilliferum* was chosen as out group. The black dots in the nodes indicate bootstrap (ML) or posterior probability (BI) values >80/0.8. The scale bar corresponds to four substitutions per 100 nucleotides positions.

TABLE S1 Hosts where Trichostomatia ciliates were registered.

MATERIAL S1 Trichostomatid families and genera.

MATERIAL S2 History of classification of subclass Trichostomatia.

VIDEO S1 Trichostomatid domestic cattle rumen ciliates under live observation.
