# Supplementary figures and images for: Trichostomatid Ciliates (Alveolata, Ciliophora, Trichostomatia) Systematics and Diversity: Past, Present, and Future
Source: Front Microbiol. 2020 Jan 15;10:2967. doi: 10.3389/fmicb.2019.02967 (PMC6974537; doi:10.3389/fmicb.2019.02967)

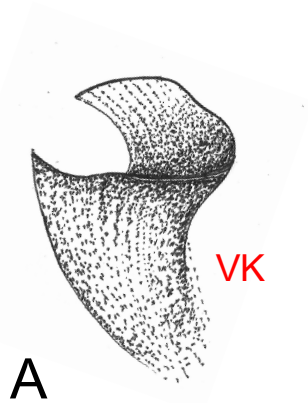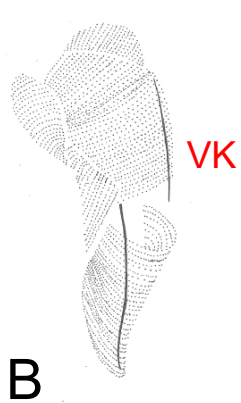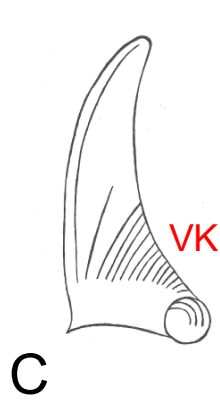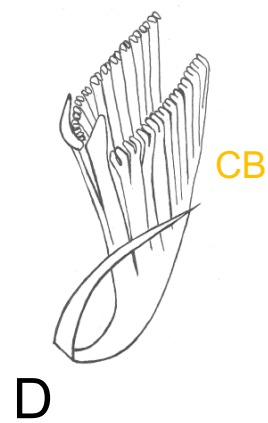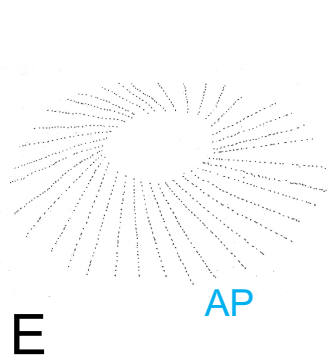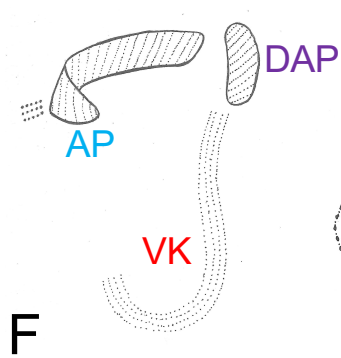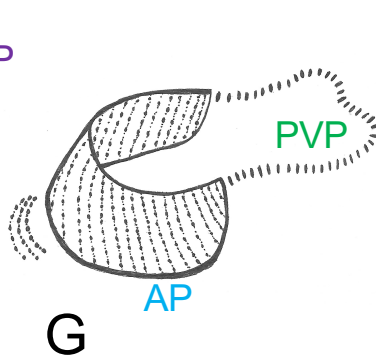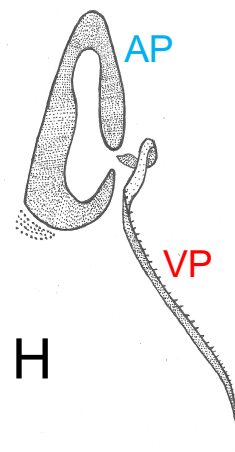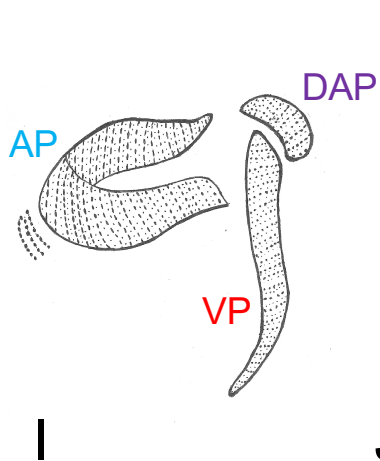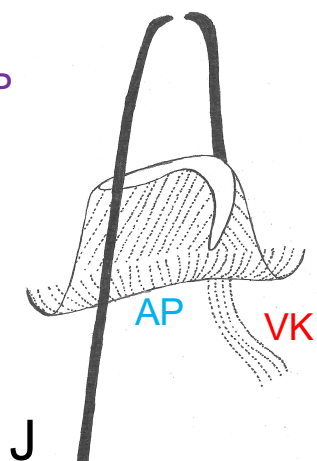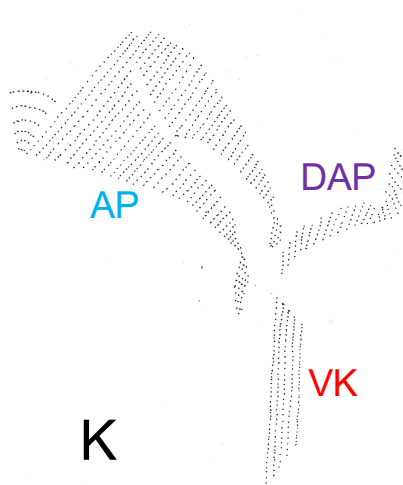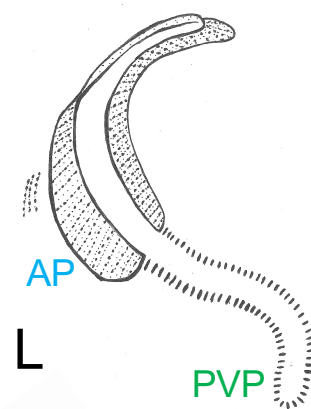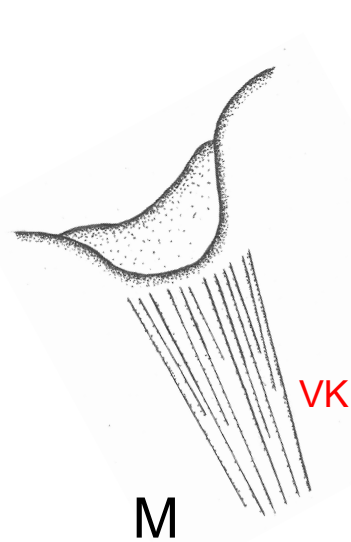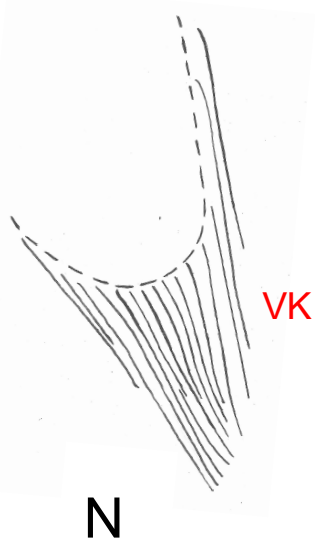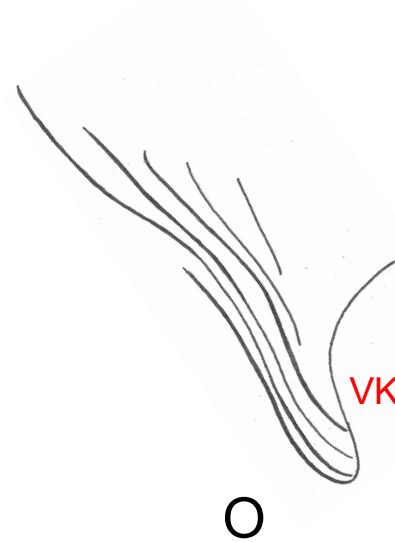

Supplement: FIGURE S1 — Oral infraciliary bands pattern of Trichostomatid ciliates. A–D. Order Vestibuliferida. A, Isotrichidae; B, Paraisotrichidae; C, Protocaviellidae; D, Protohallidae; E–L, Order Entodiniomorphida; E, Buetschliidae; F, Blepharocorythidae; G, Cycloposthiidae; H, Gichristinidae; I, Ophryoscolecidae; J, Parentodiniidae; K, Pseudoentodiniidae; L, Spirodiniidae; M–O, Order Macropodiniida; M, Amylovoracidae; N, Polycostidae; O, Macropodiniidae; AP, adoral polybrachykinety; CB, cytopharyngeal basket; DAP, dorso-adoral polybrachykinety; PVP, perivestibular polybrachykinety, and VK, vestibular kineties. [file Image_1.pdf]

Order Vestibuliferida  
Order Macropodiiniida  
Order Entodiniomorpha

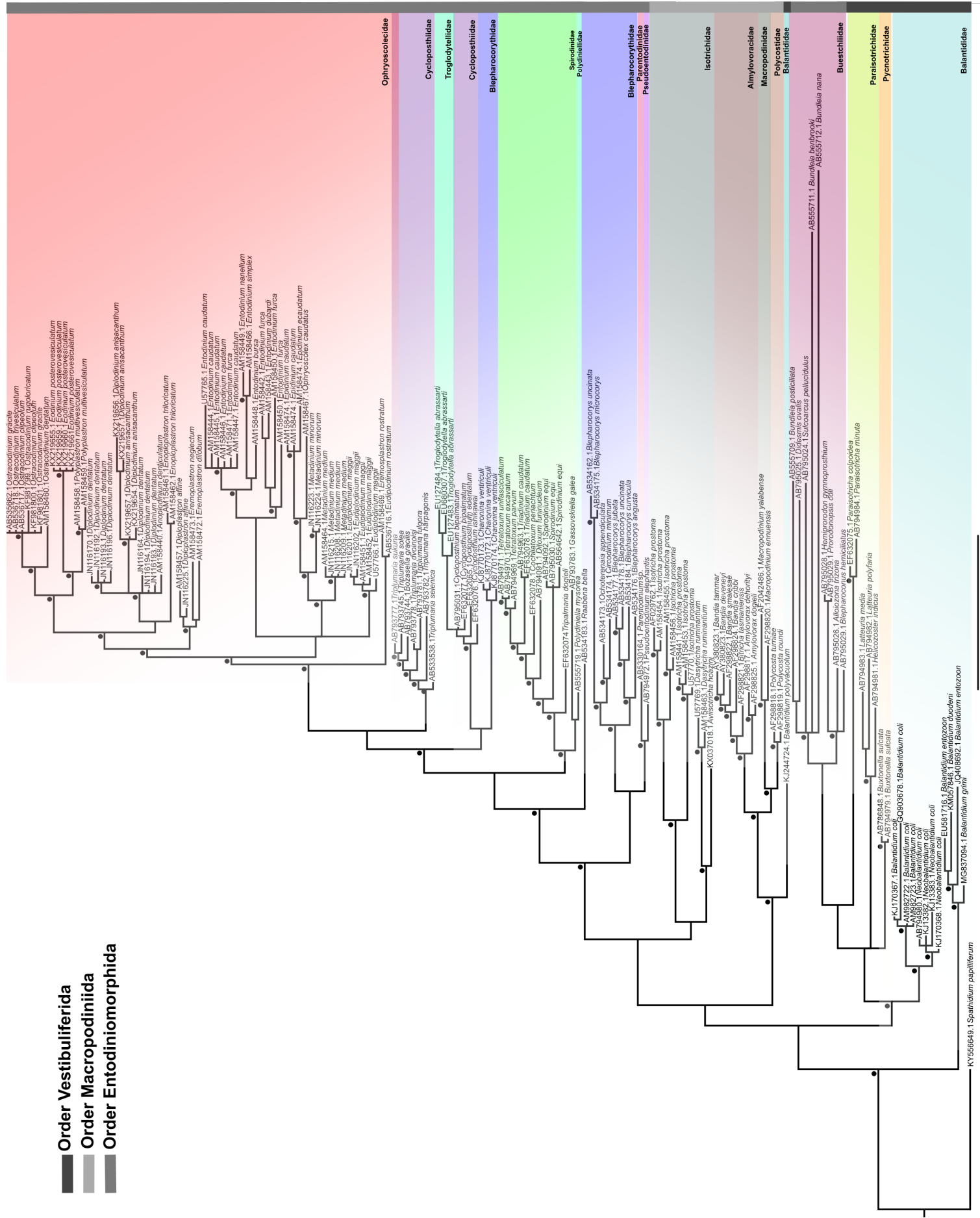

Supplement: FIGURE S2 — Phylogenetic tree of trichostomatid ciliates (Ciliophora, Litostomatea, and Trichostomatia) based on 18S rRNA gene data. Spathidium papilliferum was chosen as out group. The black dots in the nodes indicate bootstrap (ML) or posterior probability (BI) values >80/0.8. The scale bar corresponds to four substitutions per 100 nucleotides positions. [file Image_2.pdf]
