# Supplementary material for: Trichostomatid Ciliates (Alveolata, Ciliophora, Trichostomatia) Systematics and Diversity: Past, Present, and Future
Source: Front Microbiol. 2020 Jan 15;10:2967. doi: 10.3389/fmicb.2019.02967 (PMC6974537; doi:10.3389/fmicb.2019.02967)
Supplement: TABLE S1 — Hosts where Trichostomatia ciliates were registered. [file Table_1.pdf]

Supplementary Table 1. Hosts where Trichostomatia ciliates were registered

Host	Locality	Reference
Alaskan moose	Alaska	[1]
Alpaca	Bolivia	[2]
Antelope	Czech Republic	[208]
	Japan	[3]
	Kenya	[4]
	Nigeria	[5]
	South African	[6, 7, 8]
	Spain	[405]
	USA	[9, 10]
	Zambia	[11, 12]
Barbary sheep	South Korea	[13]
Bison	USA	[14]
Buffalo	Bangladesh	[15]
	Brazil	[16, 17, 18, 19]
	China	[20]
	Cuba	[21]
	Egypt	[22, 23, 24]
	Greece	[25]
	India	[26, 27, 28, 29]
	Indonesia	[30]
	Iraqi	[31]
	Japan	[32]
	Malaysia	[33]
	Nepal	[34, 35]
	Pakistan	[36]
	Philippines	[37, 38]
	South African	[39]
	Turkey	[40]
Bushpig	South African	[42]
Camel	Bahrain	[43]
	China	[41]
	Egypt	[22, 44, 45]
	Kazakhstan	[46]
	Libya	[47, 48]
	Saudi Arabia	[49]
	USA	[50]
Capybara	Bolivia	[51, 52]

	Brazil	[53, 54, 55, 56, 57, 58, 59, 60, 61, 62]
	USA	[63]
	Venezuela	[64]
Cockroach	Bulgaria	[65]
	India	[66]
Crustacean	France	[67]
Deer	Australia	[68]
	Czech Republic	[406, 407]
	Italy	[332]
	Japan	[69, 70, 71, 75]
	New Zealand	[72]
	Norway	[73]
	Poland	[74]
	USA	[76, 77, 78, 79]
Dog	China	[80]
Domestic cattle	Australia	[81]
	Belgium	[403]
	Bosnia and Herzegovina	[82]
	Brazil	[16, 17, 18, 19, 83, 84, 85, 86, 87, 88, 89, 90, 91, 92, 93, 94, 95, 96, 97, 98, 99, 100, 101, 102, 103, 104, 105]
	Canada	[106, 107]
	China	[108, 109]
	Costa Rica	[110, 111]
	Cuba	[112]
	Czech Republic	[113, 114]
	Denmark	[115]
	Eypt	[22, 23, 24]
	Finland	[116]
	Greece	[117, 118]
	Iceland	[119]
	India	[26, 120, 121, 122, 123, 124, 125, 126, 127]
	Indonesia	[30, 128]
	Iran	[129, 130]
	Iraqi	[131, 132, 133]
	Italy	[134]
	Japan	[70, 135, 136, 137,138,

		139]
	Kenya	[4, 140]
	Korea	[141]
	Libya	[48]
	Malaysia	[33]
	Mexico	[142]
	Nepal	[34]
	New Zealand	[72, 143]
	Pakistan	[144]
	Philippines	[38]
	Poland	[145, 146, 147]
	Russia	[148]
	South African	[149]
	South Korea	[150]
	Tanzania	[157]
	Thailand	[151, 152]
	Turkey	[153, 154, 155, 156, 158, 159, 160, 161, 162, 163, 164, 165, 166, 167, 168, 169]
	Uruguay	[170]
	USA	[171, 172, 173, 174, 175]
Domestic pig	Belgium	[403]
	Brazil	[176]
	Cameroon	[403]
	Central African	[403]
	Czech Republic	[403]
	China	[80]
	Greece	[25]
	India	[177]
	Kenya	[403]
	Madagascar	[403]
	USA	[50, 178, 179]
Donkey	Cyprus	[180]
	Mexico	[181]
Elephant	Central Africa	[182]
	Germany	[183]
	India	[184, 185, 186, 187]
	Japan	[188, 189]
	Kenya	[190]
	Poland	[191, 192, 193, 194]

	Republic of Congo	[182]
	Russia	[185]
	South African	[195]
	Sri Lanka	[196]
	Turkey	[197,198, 199]
	Ukraine	[183]
Elk	USA	[10, 200]
Fish	Australia	[201, 202]
	China	[203]
	Israel	[204]
	Mexico	[205]
	New Guinea	[201]
	South African	[206]
	Tuvalu Island	[207]
	Vietnam	[209]
Frog	China	[210, 211, 212]
	Czech Republic	[213]
	India	[214, 215, 216, 217, 218, 219]
	Italy	[220]
	Paquistan	[221]
	Sri Lanka	[222]
	USA	[223]
	Kenya	[4]
	Romania	[403]
	Russia	[404]
	South African	[225]
	Turkey	[226]
Giraffe	Kenya	[4]
	South African	[227]
Goat	Brazil	[228]
	Egypt	[22]
	Iceland	[119]
	Iran	[130, 229]
	Japan	[230]
	Kenya	[4]
	Nepal	[35]
	Spain	[231]
	Turkey	[232, 233, 234, 235, 236, 237, 238, 239, 240, 241]

Guanaco	Canada	[242]
Guinea pig	Brazil	[243]
	USA	[50]
Hippopotamus	Uganda	[244, 245, 246]
	Zambia	[244]
Hoatzin	USA	[247]
Horse	Albania	[248]
	Brazil	[249]
	Cyprus	[251, 252]
	Finland	[253]
	Japan	[254, 255, 256, 257, 258, 259, 260, 261, 262]
	Kyrgyzstan	[263]
	Mexico	[264,265, 266]
	New Zealand	[267]
	Russia	[224, 268, 269, 270, 271, 272, 273, 274, 275, 276, 277, 278]
	Slovakia	[279]
	Taiwan	[250]
	USA	[280, 281, 282, 283, 284, 285]
	Zambia	[256]
Human	Bangladesh	[286]
	Iran	[287]
	USA	[50]
Kulan	Slovakia	[279]
Lesser mouse deer	Malaysia	[288]
Llama	Argentina	[289]
	Bolivia	[2]
Marsupial	Australia	[290, 291, 292, 293, 294, 295, 296, 297, 298, 299]
	USA	[50]
Mule	USA	[300]
Musk- Oxen	Alaska	[1]
	Canada	[301]
	Canadian Arctic	[302]
Non-human primates	Australia	[303]
	Belgium	[304, 402, 403]
	Brazil	[176, 305]
	Cameroon	[306, 307, 308, 402, 403]

	Central Africa	[309, 310, 402, 403]
	China	[311]
	Czech Republic	[304, 312, 313, 314, 402]
	Democratic Republic of the Congo	[315, 316]
	France	[304, 402, 403]
	Gabon	[317, 318, 319]
	Germany	[304, 402, 403]
	Great Britain	[402, 403]
	Guinea-bissau	[320]
	Indonesia	[321]
	Ireland	[304, 402]
	Kenya	[402, 403]
	Japan	[322,323]
	Netherlands	[304, 402, 403]
	Nigeria	[304]
	Poland	[402, 403]
	Republic of Congo	[402, 403]
	Rwanda	[341, 324, 325, 326, 402]
	Senegal	[327, 328]
	Sierra Leone	[329]
	Slovak Republic	[304, 402]
	Spain	[304, 402, 403]
	Switzerland	[304, 402]
	Tanzania	[304, 329, 330, 331, 333, 334, 335, 402]
	Uganda	[336, 337, 338, 339, 402]
	USA	[50, 340]
	Uganda	[304]
Ostrich	USA	[50]
Reindeer	Canadian artic	[341]
	China	[342]
	Iceland	[119]
Rhinoceros	England	[343]
	Eritrea	[344]
	Kenya	[345]
	South African	[346, 347, 348, 349, 350, 351]
Salamander	China	[352, 353]
	Spain	[354]
Sheep	Alaska	[1]

	Brazil	[355, 356, 357, 358, 359, 360, 361, 362, 363, 364, 365, 366, 367, 368, 369]
	Canada	[112, 370, 371, 372, 373]
	Czech Republic	[406]
	China	[374]
	Cyprus	[375, 376, 377]
	England	[378, 379]
	Egypt	[22, 23]
	Iceland	[119]
	Iran	[380, 381, 382, 383]
	Japan	[384]
	Kenya	[4]
	Libya	[48]
	Mongolia	[385]
	New Zealand	[72, 149]
	Paquistan	[386]
	South African	[75]
	Turkey	[332, 387, 388, 389, 390, 391]
	USA	[392]
Tapir	Costa Rica	[393]
Turtle	Nicaragua	[394]
Warthog	South African	[395]
White-lipped peccary	Brazil	[396, 397]
Wild boar	Czech Republic	[403]
	Japan	[323]
Wildebeest	Kenya	[4]
	South African	[398]
Wolf	Brazil	[399]
Yak	China	[400]
Zebra	Slovakia	[279]
	South African	[401]

References

1. Dehority, B. A. (1974). Rumen ciliate fauna of Alaskan moose (*Alces americana*), musk-ox (*Ovibos moschatus*) and Dall mountain sheep (*Ovis dalli*). J. Protozool. 21:1, 26-32.

2. Del Valle, I., de la Fuente, G., and Fondevila, M. (2008). Ciliate protozoa of the forestomach of llamas (*Lama glama*) and alpacas (*Vicugna pacos*) from the Bolivian Altiplano. Zootaxa 1703:1, 62-68.

3. Kimura, Y., Tsujino, R., and Torii, H. (2017). Ciliate protozoa from the rumens of Japanese serows (*Capricornis crispus*) captured in the northern part of Shizuoka Prefecture, Japan. Mamm. Study, 42:1, 65-70.

4. Dehority, B. A., and Odenyo, A. A. (2003). Influence of diet on the rumen protozoal fauna of indigenous African wild ruminants. J. Eukaryot. Microbiol. 50, 220-223.

5. Van Hoven, W., Attwell, V. H., and Grobler, J. H. (1979). Rumen ciliate protozoa of the sable antelope Hippotragus niger. Afric. Zool. 14:1, 37-42.

6. Kleynhans, C. J. (1982). The rumen ciliates of greater kudu Tragelaphus strepsiceros (Pallas) from South Africa and Zimbabwe with a description of one new species. South African J. Zoology 17:1, 11-14.

7. Booyse, D. G., Dehority, B. A., and Myburgh, J. G. (2015). The effect of acid drinking water on rumen protozoa in the blesbok (*Damaliscus dorcas phillipsi*). Zootaxa, 4052:5, 577-582.

8. Van Hoven, W. (1983). Rumen ciliates with description of two new species from three African reedbuck species. J. Protozool. 30:4, 688-691.

9. Dehority, B. A. (1994). Rumen ciliate protozoa of the blue duiker (*Cephalophus monticola*), with observations on morphological variations lines within the species *Entodinium dubardi*. J. Eukaryot. Microbiol. 41, 103-111.

10. Dehority, B. A. (1995). Rumen Ciliates of the Pronghorn Antelope (*Antilocapra americana*), Mule Deer (*Odocoileus hemionus*), White-tailed Deer (*Odocoileus virginianus*) and Elk (*Cervus canadensis*) in the Northwestern United States. Arch. Protistenk. 146:1, 29-36.

11. Ito, A., and Tokiwa, T. (2018). Infraciliature of *Opisthotrichum janus*, *Epidinium ecaudatum*, and *Ophryoscolex purkynjei* (Ciliophora, Entodiniomorphida). Europ. J. Protistol. 62, 1-10.

12. Ito, A., Arai, N., Tsutsumi, Y., and Imai, S. (1997). Ciliate protozoa in the rumen of sassaby antelope, *Damaliscus lunatus lunatus*, including the description of a new species and form. J. Eukaryot. Microbiol. 44, 586-591.

13. Cho, H. -S., Shin, S. -S., and Park, N. -Y. (2006). Balantidiasis in the gastric lymph nodes of Barbary sheep (*Ammotragus lervia*): an incidental finding. J. Vet. Sci. 7:2, 207.

14. Towne, G. E. N. E., Nagaraja, T. G., and Kemp, K. K. (1988). Ruminal ciliated protozoa in bison. Appl. Environ. Microbiol. 54:11, 2733-2736.

15. Roy, B., Mondal, M., Talukder, M., and Majumder, S. (1970). Prevalence of *Balantidium coli* in Buffaloes at different areas of Mymensingh. J. Bangladesh Agr. Univ. 9:1, 67-72.

16. Franzolin, R., and Dehority, B. A. (1999). Comparison of protozoal populations and digestion rates between water buffalo and cattle fed an all forage diet. J. App. Anim. Res. 16:1, 33-46.

17. Franzolin, R., and Franzolin, M. H. T. (2000). População protozoários ciliados e degradabilidade ruminal em búfalos e bovinos zebuínos sob dieta à base de cana-de-açúcar. Rev. Bras. Zoot. 29:6, 1853-1861.

18. Franzolin, R., Rosales, F. P., and Soares, W. V. B. (2010). Effects of dietary energy and nitrogen supplements on rumen fermentation and protozoa population in buffalo and zebu cattle. Rev. Bras. Zoot. 39:3, 549-555.

19. Ríspoli, T. B., Rodrigues, I. L., Martins Neto, R. G., Kazama, R., Prado, O. P. P., Zeoula, L. M., and Arcuri, P. B. (2009). Ruminal ciliate protozoa of cattle and buffalo fed on diet supplemented with monensin or extracts from propolis. Pesq. Agr. Bras. 44:1, 92-97.

20. Jiaju, R. (1984). Ciliate protozoa in the rumen of chinese water buffalo, *Bubalus bubalis* Linnaeus. J. Nanjing Agr. Univ.

21. González, N., Galindo, J., Aldana, A. I., and Marrero, Y. (2007). Identificación y comparación de géneros de protozoos presentes en el líquido ruminal de búfalos de río y bovinos Cebú alimentados con forrajes. Nota técnica. Rev. Cub. Cienc. Agr. 41:4.

22. Baraka, T. A. (2012). Comparative studies of rumen pH, total protozoa count, generic and species composition of ciliates in camel, buffalo, cattle, sheep and goat in Egypt. J. Am. Sci., 8:2, 448-462.

23. Selim, H. M., Imai, S., Yamato, O., El Kabbany, A., Kiroloss, E., and Maeda, Y. (1996). Comparative study of rumen ciliates in buffalo, cattle and sheep in Egypt. J. Vet. Med. Sci., 58, 799-801.

24. Sultan, K., Khalafalla, R. E., and Elseify, M. A. (2013). Preliminary investigation on *Buxtonella sulcata* (Jameson, 1926) (Ciliophora: Trichostomatidae) in Egyptian Ruminants. B. S. Vet. Med. J. 22, 91-94.

25. Himonas, C. A., Antoniadou-Soteriadou, K. S., Sotiraki, S. T., and Papazahariadou, M. G. (1998). Intestinal protozoa of animals in the Macedonia region of Greece. Bull. Hell. Vet. Med. Soc. 49:4, 300-306.

26. Ganai, A., Parveen, S., Kaur, D., Katoch, R., Yadav, A., Godara, R., and Ahamed, I. (2015). Incidence of *Buxtonella sulcata* in bovines in RS Pura, Jammu. J. Parasitic Diseases 39:3, 446-447.

27. Kumar, B., Maharana, B. R., Prashad, A., Joseph, J. P., and Patel, B. R. (2017). Incidence of *Buxtonella sulcata* in Jaffrabadi buffaloes of south-western Gujarat, India. Buffalo Bull. 36:4, 623-628.

28. Singh, K. M., Tripathi, A. K., Pandya, P. R., Rank, D. N., Kothari, R. K., and Joshi, C. G. (2011). *Dasytricha* Dominance in Surti Buffalo Rumen Revealed by 18S rRNA Sequences and Real-Time PCR Assay. Current Microbiol. 63:3, 281-288.

29. Singh, K. M., Pandya, P. R., Tripathi, A. K., Patel, G. R., Parnerkar, S., Kothari, R. K., and Joshi, C. G. (2013). Molecular diversity of protozoa in rumen of Indian buffalo (*Bubalus bubalis*). Agr. Res. 2:4, 360-366.

30. Imai, S. (1985). Rumen ciliate protozoal faunae of Bali cattle (*Bos javanicus domesticus*) and water buffalo (*Bubalus bubalis*) in Indonesia, with the description of a new species, *Entodinium javanicum* sp. nov. Zool. Sci. 2:4, 591-600.

31. Azhar, C., Karawan, M., and Jadaan, M. A. Diagnostic study of gastrointestinal parasites in buffaloes of Diwaniya Province. (2017). Basrah J. Vet. Res. 16:1, 298-312.

32. Imai, S., Fujita, J., and Ogimoto, K. (1981). Rumen ciliate protozoal fauna of water buffalo, *Bubalus bubalis* (Linnaeus), in Okinawa, Japan. Bull. Nippon Vet. Zootech. Coll.

33. Imai, S., Abdullah, N., Ho, Y. W., Jalaludin, S., Hussain, H. Y., Onodera, R., and Kudo, H. (1995). Comparative study on the rumen ciliate populations in small experimental herds of water buffalo and Kedah Kelantan cattle in Malaysia. Anim. F. Sci. Technol. 52:3-4, 345-351.

34. Adhikari, B. B., Rana, H. B., Sultan, K. M., Devkotal, B., Nakao, T., Kobayashi, K., and Dhakal, I. P. (2013). Prevalence of *Buxtonella sulcata* in water buffaloes and cows in Chitwan Valley, southern Nepal. Jpn. J. Vet. Parasitol. 11:2, 55-60.

35. Gurung, Y. B., Parajuli, N., Miyazaki, Y., Imai, S., and Kobayashi, K. (2002). Rumen ciliate faunae of water buffalo (*Bubalus bubalis*) and goat (*Capra hircus*) in Nepal. J. Vet. Med. Sci. 64:3, 265-267.

36. Tarrar, M.A., Khan, M.S., Pervez, K., Ashraf, K., Khan, J.A., and Rehman, Z.U. (2008). Detection and chemotherapy of *Balantidium coli* in buffaloes around Lahore, Pakistan. Pak. J. Agric. Sci. 45, 163-166.

37. Dianso, J. A., Garcia, G. G., Belotindos, L. P., and Mingala, C. N. (2018). Molecular identification of *Buxtonella sulcata* from associated-diarrhea in water buffaloes (*Bubalus bubalis*) in the Philippines. Ann. Parasitol. 64:2, 93-100.

38. Shimizu, M., Kinoshita, M., Fujita, J., and Imai, S. (1983). Rumen ciliate protozoal fauna and composition of the zebu cattle, *Bos indicus*, and water buffalo, Bubalus bubalis, in Philippines. Bull. Nippon Vet. Zootech. Coll.

39. Booyse, D. G., Dehority, B. A., and Reininghaus, B. J. Ö. R. N. (2014). Rumen ciliates in the African (Cape) buffalo (*Syncerus caffer caffer*) living in the vicinity of the Orpen Gate entrance into Kruger National Park, South Africa. Zootaxa 3846:1, 138-144.

40. Gürelli, G. (2018). Infraciliature of *Eudiplodinium dilobum*, *E. rostratum*, and *E. maggii* (Ciliophora, Entodiniomorphida). Comm. J. Biol. 2:2, 16-18.

41. Imai, S., and Rung, G. (1990). Ciliate protozoa in the forestomach of the bactrian camel in Inner-Mongolia, China. Jpn. J. Vet. Sci. 52:5, 1069-1075.

42. Van Hoven, W., and Gilchrist, F. M. (1991). First record of ciliated protozoan endocommensals in the gut of bush pig. South African J. Wildlife 21:1, 28-29.

43. Abubakr, M. I., Nayel, M. N., Fadlalla, M. E., Abdelrahman, A. O., Abuobeida, S. A., and Elgabara, Y. M. (2000). Prevalence of gastrointestinal parasites in young camels in Bahrain. Revue D'elevage et de Medicine Veterinaire des Pays Tropicaux. 53:3, 267-272.

44. Kubesy, A. A., and Dehority, B. A. (2002). Forestomach ciliate Protozoa in Egyptian dromedary camels (*Camelus dromedarius*). Zootaxa 51:1, 1-12.

45. Selim, H. M., IMAI, S., YAMATO, O., MIYAGAWA, E., and MAEDE, Y. (1996). Ciliate protozoa in the forestomach of the dromedary camel, (*Camelus dromedarius*), in Egypt, with description of a new species. J. Vet. Med. Sci. 58:9, 833-837.

46. Dogiel, V. (1928). La faune d'infusoires habitant l'estomac du buffle et du dromadaire. Ann. Parasit. Hum. Comp. 6:3, 323-338.

47. Gürelli, G., and Mohamed, A. R. A. (2018). Ciliated Protozoan Fauna in the Forestomach of Dromedary Camels (*Camelus dromedarius*) in Libya. Zootaxa 4434:3, 429-440.

48. Selim, H. M., Imai, S., El Sheik, A., Okamoto, E., Miyagawa, E., and Maede, Y. (1999). Rumen ciliate protozoal fauna of native sheep, Friesian cattle and dromedary camel in Libya. J. Vet. Med. Sci. 61:3, 303-305.

49. Al-Tayib, O. (2014). Zoonotic balantidiasis in camel from Saudi Arabia. Scholars Academic Journal of Biosciences 2:7, 445-447.

50. Hegner, R. (1934). Specificity in the genus *Balantidium* based on size and shape of body and macronucleus, with descriptions of six new species. American J. Epidem. 19:1, 38-67.

51. Ito A., and Imai S. (2000a). Ciliates from the cecum of capybara (*Hydrochoerus hydrochaeris*) in Bolivia 1. The families Hydrochoerellidae n. fam., Protohallidae, and Pycnotrichidae. Europ. J. Protistol. 36, 53-84.

52. Ito A., and Imai S. (2000b). Ciliates from the cecum of capybara (*Hydrochoerus hydrochaeris*) in Bolivia 2. The family Cycloposthiidae. Europ. J. Protistol. 36, 169-200.

53. Batisse A. (1965). Nouvelle contribution a l’etude des infusoires parasites du coecum de l’hydrocheire (*Hydrocheirus capybara* L.). II. *Muniziella cunhai*, Da Fonseca, représentant neotropical de la famille des Pycnotrichidae. Protist 1, 41-51.

54. Batisse A. (1966). Quelques infusoires holotriches parasites du coecum de l’hydrocheire (*Hydrocheirus capybara* L.). Protist 2, 39-52.

55. Cedrola, F., Fregulia, P., D'Agosto, M., Dias, R. J. P. (2018). Intestinal ciliates of Brazilian Capybara (*Hydrochoerus hydrochaeris* L.). Acta Protozool. 57, 61-67.

56. Cunha, A. M. (1914). Sobre os ciliados intestinais dos mamiferos. Mem. Inst. Oswaldo Cruz 6, 212-216.

57. Cunha, A. M. (1915). Sobre os ciliados intestinaes dos mamiferos. Mem. Inst. Oswaldo Cruz 7, 139-145.

58. Cunha, A. M., and Muniz, J. (1925). Contribuição para o conhecimento dos ciliados parasitos dos mamíferos do Brasil. Sci. Med. 3, 732-747.

59. Cunha A. M., and Muniz J. (1927a). Sur quelques ciliés parasites des mammifères du Brésil. C. R. Séanc. Soc. Biol. Ses. Fil. 96, 492-493.

60. Cunha A. M., and Muniz J. (1927b). Trois nouvelles espèces du *Cycloposthium*. C. R. Séanc. Soc. Biol. Ses. Fil. 96, 494-496.

61. Fonseca F. (1939). Protozoarios parasitas. I. Ciliado gigante, *Muniziella cunhai*, gen. n., sp. n., parasita de *Hydrochoerus capybara* (Holotricha, Pycnothrichidae). Mem. Inst. Butantan 12, 165-172.

62. Hollande A., and Batisse A. (1959). Contribution al'etude des infsoires parasites du coecum de I'hydrocheire (*Hydrocheirus capybara* L.). I. La famille des Cycloposthiidae. Mem. Inst. Oswaldo Cruz 57, 1-16.

63. Dehority, B. A. (1987). Rumen ophryoscolecid protozoa in the hindgut of the capybara (*Hydrochoerus hydrochaeris*). J. Protozool. 34, 143-145.

64. McLure, M. T. (1976). The cecal ciliates of the Venezuelan capybara (*Hydrochoerus hydrochaeris* and *H. isthmius*). Trans. Am. Microsc. Soc. 95, 268.

65. Nedelchev, S., Pilarska, D., Takov, D., and Golemansky, V. (2013). Protozoan and Nematode Parasites of the American Coakroach *Periplaneta americana* (L.) from Bulgaria. Acta Zool. Bulg. 65, 403-408.

66. Ghosh, E. (1922). On a New Ciliate, *Balantidium ovatum*, sp. nov., an Intestinal Parasite in the Common Cockroach (*Blatta americana*). Parasitology, 14:3-4, 371.

67. Poisson, R. (1921). Sur un infusoire du genre *Balantidium*, parasite du tube digestif d'Orchestia littorea Mont. C. R. Seanc. Soc. Biol. Fil. 84:333-335.

68. Dehority, B. A. (1997). Rumen ciliate protozoa in Australian red deer (*Cervus elaphus* L.). Arch. Protistenk. 148:1-2, 157-165.

69. Imai, S., Matsumoto, M., Watanabe, A., and Sato, H. (1993). Rumen ciliate protozoa in Japaneese sika deer (*Cervus nippon centralis*). Anim. Sci. Technol. 64, 578-583.

70. Imai, S., Matsumoto, M., Watanabe, A., and Sato, H. (2002). Establishment of a Spinated Type of *Diplodinium rangiferi* by Transfaunation of the Rumen Ciliates of Japanese Sika Deer (*Cervus nippon*) centralist to the Rumen of Two Japanese Shorthorn Calves (*Bos taurus taurus*). J. Eukaryot. Microbiol. 49:1, 38-41.

71. Ito, A., Imai, S., and Ogimoto, K. (1993). Rumen ciliates of Ezo deer (*Cervus nippon yesoensis*) with the morphological comparison with those of cattle. J. Vet. Med. Sci. 55:1, 93-98.

72. Kittelmann, S., and Janssen, P. H. (2011). Characterization of rumen ciliate community composition in domestic sheep, deer, and cattle, feeding on varying diets, by means of PCR-DGGE and clone libraries. FEMS Microbiol. Ecol. 75:3, 468-481.

73. Aagnes, T. H., Sormo, W. and Mathiesen, S. D. 1995. Ruminal microbial digestion in free-living, in captive lichen-fed and in starved rein deer (*Rangifer tarandus tarandus*) in winter. Appl. Environ. Microbiol. 61:583-591.

74. Tomczuk, K., Szczepaniak, K., Grzybek, M., Studzińska, M., Demkowska-Kutrzepa, M., Roczeń-Karczmarz, M., and Bojar, W. (2017). Internal parasites in roe deer of the Lubartów Forest Division in postmortem studies. Med. Wet. 73:11, 726-730.

75. Ito, A., Imai, S., Ogimoto, K. (1993). Rumen Ciliates of Ezo Deer (*Cervus nippon yesoensis*) with the Morphological Comparison with those of Cattle. J. Vet. Med. Sci. 55:1, 93-98.

76. Dehority, B. A., Demarais, S., and Osborn, D. A. (1999). Rumen Ciliates of White-tailed Deer (*Odocoileus virginianus*), Axis Deer (*Axis axis*), Sika Deer (*Cervus nippon*) and Fallow Deer (*Dama dama*) from Texas. J. Eukaryot. Microbiol. 46:2, 125-131.

77. MacLennan, R. F. (1935). Ciliates from the stomach of mule-deer. Trans. Am. Microsc. Soc. 54:3, 181-188.

78. Pearson, H. A. (1965). Rumen organisms in white-tailed deer from south Texas. J. Wildlife Management, 493-496.

79. Pearson, H. A. (1969). Rumen microbial ecology in mule deer. Appl. Environ. Microbiol. 17:6, 819-824.

80. Yin, D. -M., Lv, C. -C., Tan, L., Zhang, T. -N., Yang, C. -Z., Liu, Y., and Liu, W. (2015). Prevalence of *Balantidium coli* infection in sows in Hunan province, subtropical China. Trop. A. H. Production, 47:8, 1637-1640.

81. Klieve, A. V., Holroyd, R. G., Turner, A. F., and Lindsay, J. A. (1998). Rumen bacterial and protozoal populations in cattle being relocated in tropical Queensland. Australian J. Agr. Res. 49:7, 1153-1160.

82. Omeragić, J., and Crnkić, Ć. (2015). Diarrhoea in cattle caused by *Buxtonella sulcata* in Sarajevo area. Veterinaria 64:2, 50-54.

83. Cedrola, F., Senra, M. V. X., D'Agosto, M., and Dias, R. J. P. (2017). Phylogenetic Analyses Support Validity of Genus *Eodinium* (Ciliophora, Entodiniomorphida, Ophryoscolecidae). J. Eukaryot. Microbiol. 64:2, 242-247.

84. Coalho, M. R., Nogueira Filho, J. C. M., Cunha, J. A., and de Lima, C. G. (2003). Estudo dos protozoários ciliados em bovinos consumindo dietas com diferentes níveis de proteína não degradável no rúmen. Acta Scient. Anim. Sci. 25:1, 193-199.

85. Costa, F. A. A., Fernandes, L. B., Garcia, V. P., Soares, W. V. B., and Franzolin, R. (2017). Degradability of grasses, ruminal fermentation and protozoa in beef cattle on diet with different additives. Rev. Bras. S. Prod. Anim. 18:2, 269-281.

86. D'Agosto, M. D., Carneiro, M. E., Netto, C. M. M., and Arcuri, P. B. (1996). Avaliação dos ciliados do rúmen de bovinos mantidos com duas dietas (in Portuguese). Arq. Bras. Med. Vet. Zoo. 48:353-361.

87. D'Agosto, M., and Guedes, P. D. M. (2000). Characterization of beef cattle rumen ciliate (Protista, Ciliophora) populations in Minas Gerais State, Brazil. Rev. Bras. Zooc. 2:1, 81-90.

88. D'Agosto, M., and Guedes, P. M. D. M. (2001). Associative behaviour and antagonism of bovine rumen ciliate (Protista, Ciliophora) from Zona da Mata, Minas Gerais State, Brazil. Rev. Bras. Zool. 18:4, 1123-1126.

89. D'Agosto, M., and Santa-Rosa, M. R. (1998). Influência do hospedeiro no perfil populacional e nas populações de ciliados do rúmen de bovinos. Rev. Bras. Zool. 15:2, 389-396.

90. D'Agosto, M., and Salvio, G. M. M. (2001). Ciliados nas cavidades do estômago de bovinos. Arq. Bras. Med. Vet. Zoot. 53:6, 686-690.

91. D'Agosto, M., Siqueira, I. C. V., and Santo, N. B. E. (2001). Comportamento e distribuição de protozoários ciliados (Protista, Ciliophora) no rúmen e no retículo de bovinos submetidos ao jejum. Rev. Bras. C. Vet. 8:1, 16-18.

92. Dehority, B. A. (1986). Rumen ciliate fauna of some Brazilian cattle: Occurrence of several ciliates new to the rumen, including the Cycloposthid *Parentodinium africanum*. J. Protozool. 33, 416-421.

93. dos Santos, A. C. R., Magalhães, D. Q., Azevedo, R. A., Vieira, I. L. N., França, D. E., Geraseev, L. C., and Duarte, E. R. (2015). Efeito da inclusão da torta de macaúba na população de protozoários do rúmen de vacas leiteiras. Arq. Bras. Med. Vet. Zootec. 67:6, 1653-1659.

94. Duarte, E. R., Abrão, F. O., Oliveira Ribeiro, I. C., Vieira, E. A., Nigri, A. C., Silva, K. L., and Gerasev, L. C. (2018). Rumen protozoa of different ages of beef cattle raised in tropical pastures during the dry season. J. App. Anim. Res. 46:1, 1457-1461.

95. Martinele, I., Detoni, M., Rust, N. M., D'Agosto, M. (2007). Protozoários ciliados (Protista, Ciliophora) no conteúdo do rúmen e do retículo de bovinos. Rev. Bras. Zooc. 9, 63-66.

96. Martinele, I., Siqueira-Castro, I. C. V., and D'Agosto, M. (2008). Rumen ciliate protozoa in cattle fed elephant-grass and two concentrate levels. Rev. Bras. S. Prod. Anim. 9:1, 74-81.

97. Nigri, A. C. A., Ribeiro, I. C. O., Vieira, E. A., Silva, M. L. F., Virgínio-Júnior, G. F., Abrão, F. O., and Duarte, E. R. (2017). Population of Protozoa in zebu steers fed with or without bulk. Arqu. Bras. Med. Vet. Zoot. 69:5, 1339-1345.

98. Nogueira Filho, J. C. M., de Oliveira, M. E. M., de Souza Ablas, D., Titto, E. A. L., de Toledo, L. R. A., and de Oliveira, T. S. B. M. (2000). Fauna ciliada do rúmen de zebuínos e bubalinos em Pirassununga, São Paulo, Sudeste do Brasil. Acta Scientiarum Anim. Sci. 22, 663-668.

99. Nogueira Filho, J. C. M., de Oliveira, M. E. M., Veiga, J. S. M., and de Souza Lucci, C. (1983). Observações pertinentes à instalação da fauna de protozoários ciliados no rúmen de bezerros de raça Holandesa (*Bos taurus*, L.), criados em Pindamonhangaba, SP, Brasil. Braz J Vet Res Anim Sci. 20:2, 177-182.

100. Nogueira Filho, J. C. M., de Sousa Lucci, C., de Oliveira, M. E. M., Melotti, L., Valvasori, E., de Lima, C. G., and da Cunha, J. A. (1990). Influência da soja fornecida crua, tostada ou como farelo, na composição de rações para bovinos, sobre o número e gêneros de protozoários ciliados do rúmen. Braz J Vet Res Anim Sci. 27:1, 123-127.

101. Nogueira Filho, J. C. M., Lucci, C. D. S., Melotti, L., Oliveira, M. E. M. de, Lima, C. G. de, and Cunha, J. A. da. (1992). Contagens diferenciais de protozoários ciliados em rúmen de bovinos arraçoados com capim elefante Napier (Pennisetum purpureum Schum), em vários estádios de crescimento vegetativo. Braz J Vet Res Anim Sci. 29:2, 215.

102. Nogueira Filho, J. C. M., Oliveira, M. E. M. de, Franzolin Neto, R., Schalch, E., and Velloso, L. (1991). Avaliação dos protozoários ciliados no rúmen de búfalos (*Bubalus bubalis* L.) e bovinos (*Bos indicus* L.) em regime de confinamento. Braz J Vet Res Anim Sci. 28:2, 243.

103. Rossi, M. F., Dias, R. J. P., Senra, M. V. X., Martinele, I., Soares, C. A. G., and D'Agosto, M. (2015). Molecular Phylogeny of the Family Ophryscolecidae (Ciliophora, Litostomatea) Inferred from18SrDNA Sequences.J Eukaryot Microbiol. 62:5, 584-590.

104. Rossi, M. F., Martinele, I., and D'Agosto, M. (2013). Quantitative and differential analysis of ciliate protozoa in rumen content samples filtered before and after fixation. Rev. Bras. Zootec. 42:11, 831-834.

105. Rossi, M., Cedrola, F., Dias, R. J. P., Martinele, I., and D'Agosto, M. (2016). Improved silver carbonate impregnation method for rumen ciliate protozoa. Rev. Bras. Zooc. 17:1.

106. Imai, S., Han, S. S., Cheng, K. J., and Kudo, H. (1989). Composition of the rumen ciliate population in experimental herds of cattle and sheep in Lethbridge, Alberta, Western Canada. Can. J. Microbiol., 35:7, 686-690.

107. Zhou, M., Hünerberg, M., Beauchemin, K. A., McAllister, T. A., Okine, E. K., and Guan, L. L. (2012). Individuality of ruminal methanogen/protozoa populations in beef cattle fed diets containing dried distillers' grain with solubles. Acta Agric. Scand. A Anim. Sci. 62:4, 273-288.

108. Rung, G., and Imai, S. (1989). Rumen ciliate protozoal fauna and composition of the cattle in Nei-Mongol, China. Acta Vet. Zootech. Sinica. 20, 168-175.

109. Su, N. R., Xiang Hua, Z. H. A. I., Zhu, S., and Imai, S. (2000). Rumen ciliated protozoan fauna of the yak (*Bos grunniens*) in China with the description of *Entodinium monuo* n. sp.. J. Eukaryot. Microbiol. 47:2, 178-182.

110. Jiménez-Rocha, A. E., Montenegro-Hidalgo, V. M., Hernández-Gamboa, J., Dolz-Wiedner, G., Maranda, L., Galindo-Badilla, J. R., and Schnieder, T. (2007). Dynamics of infections with gastrointestinal parasites and *Dictyocaulus viviparus* in dairy and beef cattle from Costa Rica. Vet. Parasitol. 148:3/4, 262-271.

111. Jiménez, A. E., Fernández, A., Alfaro, R., Dolz, G., Vargas, B., Epe, C., and Schnieder, T. (2010). A cross-sectional survey of gastrointestinal parasites with dispersal stages in feces from Costa Rican dairy calves. Vet. Parasitol. 173:3/4, 236-246.

112. Sedloev, N., and Nieto, N. (1985). Genera *Ophryoscolex* in rumen contents of cattle in Cuba. Short Communication. Rev. Salud Anim..

113. Grim, J. N., Jirků-Pomajbíková, K., and Ponce-Gordo, F. (2015). Light microscopic morphometrics, ultrastructure, and molecular phylogeny of the putative pycnotrichid Ciliate, *Buxtonella sulcata*. Eur. J. Protistol. 51:5, 425-436.

114. Jirovec, O. (1933). Beobachtungen tiber die Fauna des Rinderpansen. Z. Parasitenkd. 5, 584-591.

115. Henriksen, S. A. (1977) *Buxtonella sulcata*, an intestinal ciliate of apparently frequent occurrence in Danish cattle (author's transl). Nord. Vet. Med. 29:10, 452-457.

116. Tapio, I., Fischer, D., Blasco, L., Tapio, M., Wallace, R. J., Bayat, A. R.,Vilkki, J. (2017). Taxon abundance, diversity, co-occurrence and network analysis of the ruminal microbiota in response to dietary changes in dairy cows. PLoS One. 12, 7.

117. Diakou, A., Papadopoulos, E., Haralabidis, S., Papachristou, F., Karatzias, H., and Panousis, N. (2005). Prevalence of parasites in intensively managed dairy cattle in Thessaloniki Region, Greece. Cattle Pract. 13, 51-54.

118. Diakou, A., and Papadopoulos, E. (2018). Prevalence of gastrointestinal parasites of cattle in Greece. J. Hellenic Vet. Med. Soc. 53:4, 304-309.

119. De la Fuente, G., Skirnisson, K., and Dehority, B. A. (2006). Rumen ciliate fauna of Icelandic cattle, sheep, goats and reindeer. Zootaxa. 1377, 47-60.

120. Edith, R., Balagangatharathilagar, M., Gomathinayagam, S., and Roy, P. (2018). Incidence of *Buxtonella sulcata* infection in cattle from organized and unorganized dairy farms in tamil nadu. Int. J. Sci. Environ. Technol. 7:1, 133-137.

121. Kulkarni, S. A. (2013). A new species of protozoan ciliate *Entodinium conicospinum* (sp. nov) from the rumen of Indian cattle (*Bos indicus*). Gold. Res. Thoughts. 3, 1.

122. Murthy, C. K., and D'Souza, P. E. (2016). Prevalence of gastrointestinal parasites in bovines in Bangalore district, Karnataka. J. Parasit. Dis. 40:3, 630-632.

123. Sanghai, P. K. (2018). Occurrence of ciliate protozoa *Epidinium bulbiferum* (Dogiel 1927) and *Epidinium hamatum* (Schulze 1924) from the rumen of cattle (*Bos indicus*). Int. J. Zool. Stud. 3, 374-77.

124. Sanghai, P. K., and Kshirsagar, H. S. (2015). The occurrence of the rumen ciliate *Enoploplastron triloricatum* (Dogiel 1925) from the rumen of *Bos indicus* in India. Ijpaz, 3:2, 176-180.

125. Kofoid, C. A. and Maclennan, R. F. (1930). Ciliates from *Bos indicus* Linn. I. The genus *Entodinium* Stein. Univ. Calif. Publ. Zool. 33, 471-544.

126. Kofoid, C. A and Maclennan, R. F. (1932). Ciliates from *Bos indicus* Linn. II. A revision of *Diplodinium* Schuberg. Univ. Calif. Publ. Zool. 37, 53-152.

127. Kofoid, C. A and Maclennan, R. F. (1933). Ciliates from Bos indicus Linn. III. *Epidinium* Crawley, *Epiplastron* gen. nov., and *Ophryoscolex* Stein. Calif. Publ. Zool. 39, 1-34.

128. Wisesa, I. B. G. R., Siswanto, F. M., Putra, T.A., Oka, I. B. M., Suratma, N. A. (2015). Prevalence of *Balantidium* sp. in Bali cattle at different areas of Bali. Int. J. Agric. For. Life Sci.1, 49-53.

129. Hasheminasab, S. S., Moradi, P., Talvar, H. M., Wright, I., and Darbandi, M. S. (2015). *Buxtonella* spp. like infection in cattle in Sanandaj province, Iran. Ann. Parasitol. 61, 4.

130. Talar, S., Arbabi, M., and Talari, M. R. (2004). Ciliates of the rumen of domestic ruminants in Kashan. Arch. Razi Ins. 57, 121-126

131. Al-Bakri, H. S., Suliman, E. G., and Al-Saffar, T. M. (2010). Prevalence of intestinal ciliate *Buxtonella sulcata* in cattle in Mosul. Iraqi. J. Vet. Sci. 24:1, 27-30.

132. Al-Mayah, K. S., and Al-Zubaidi, M. T. (2011). Prevalence of *Buxtonella sulcata* in neonatal and young calves in Al-Nasir station and some regions in Baghdad (Al-Shuala and Gazaliya). Iraqi J. Sci. 52:4, 420-424.

133. Hussin, A., and Al-Samarai, F. (2016). Prevalence of *Balantidium coli* in cattle and cattle breeders in some regions of Baghdad in Iraq. Bangladesh J. Anim. Sci. 45:2, 30.

134. Moriggi, M. (1941) Ricerche sugli infusori dei ruminanti italiani. Arch. Zool. Ital. 29, 369-412.

135. Ito, A., and Imai, S. (1998). Infraciliary bands in the rumen ophryoscolecid ciliate *Ostracodinium gracile* (Dogiel, 1925), observed by light microscopy. J. Eukaryot. Microbiol. 45:6, 628-636.

136. Ito, A., and Imai, S. (2003). Light microscopical observation of infraciliary bands of *Eodinium posterovesiculatum* in comparison with *Entodinium bursa* and *Diplodinium dentatum*. J. Eukaryot. Microbiol. 50:1, 34-42.

137. Ito, A., and Imai, S. (2005). Infraciliature and morphogenesis in three rumen *Diplodinium* ciliates, *Diplodinium polygonale*, *Diplodinium leche*, and *Diplodinium nanum*, observed by light microscopy. J. Eukaryot. Microbiol. 52:1, 44-51.

138. Ito, A., Imai, S. and Ogimoto, K. (1994). Rumen ciliate composition and diversity of Japanese beef black cattle in comparison with those of Holstein-Friesian cattle. J. Vet. Med. Sci. 56:707-714.

139. Ito, A., Miyazaki, Y., and Imai, S. (2001). Light microscopic observations of infraciliature and morphogenesis in six species of rumen *Ostracodinium* ciliates. J. Eukaryot. Microbiol., 48:4, 440-448.

140. Imai, S. (1988). Ciliate Protozoa in the Rumen of Kenyan Zebu Cattle, *Bos taurus* indicus, with the Description of Four New Species 1. J. Protozool. 35:1, 130-136.

141. Kang, Y. B., Chung, K. S., Kim, J. S., and Kim, D. H. (1989). Identification and population density of major ciliates in rumen of Korean native cattle. Korean Journal of Veterinary Public Health (Korea R.).

142. Imai, S. and Kinoshita, M. (1997). Comparison of rumen ciliate compositions among Hereford, Holstein and zebu cattle in Mexico. Rev. Soc. Mex. Hist. Nut. 47, 85-91.

143. Kittelmann, S., Devente, S. R., Kirk, M. R., Seedorf, H., Dehority, B. A., and Janssen, P. H. (2015). Phylogeny of intestinal ciliates, including *Charonina ventriculi*, and comparison of microscopy and 18S rRNA gene pyrosequencing for rumen ciliate community structure analysis. Appl. Environ. Microbiol. 81:7, 2433-2444.

144. Bilal, C. Q., Khan, M. S., Avais, M., Ijaz, M., and Khan, J. A. (2009). Prevalence and chemotherapy of *Balantidium coli* in cattle in the River Ravi region, Lahore (Pakistan). Vet. Parasitol. 163:1/2, 15-17.

145. Kowalik, B., Majewska, M. P., Pająk, J. J., and Skomiał, J. (2015). Effect of the preparation Ruchamax in diets for heifers on the population of ciliates, rumen fermentation, and biochemical parameters of blood. Med. Weter. 71:9, 578-582.

146. Studzińska, M., Bogucki, J., Demkowska-Kutrzepa, M., Roczeń-Karczmarz, M., Szczepaniak, K., Junkuszew, A., and Tomczuk, K. (2018). Gastrointestinal parasites in calves in small and middle-sized farms of South-east Poland. Med. Weter. 74:8, 520-525.

147. Tomczuk, K.. F., Kurek, L., Stec, A., Studzinska, M., and Mochol, J. (2005). Incidence and clinical aspects of colon ciliate *Buxtonella sulcata* infection in cattle. Bull. Vet. Inst. Pulawy. 49:1, 29-33.

148. Shibitov, S. K., and Safiullin, R. T. (2016). Prevalence of *Buxtonella sulcata* (Jameson, 1926) among cattle in the Kurgan region. Rossiĭskiĭ Parazitol. Z. 4, 509-514.

149. Fantham, H. B. (1930). Some parasitic protozoa found in South Africa. XIII. South African J. Sci. 27, 376-390.

150. Hong, K. O., and Youn, H. J. (1995). Incidence of *Buxtonella sulcata* from cattle in Kyonggi-do. Korean J. Parasitol. 33:2, 135-138.

151. Imai, S. (1984). New rumen ciliates, *Polymorphella bovis* sp. n. and *Entodinium longinucleatum* forma *spinolobum* fn, from the zebu cattle in Thailand. Jpn. J. Vet. Sci. 46:3, 391-395.

152. Imai, S., and Ogimoto K. (1983). *Parabundleia ruminantium* gen. n., sp. n., *Diplodinium mahidoli* sp. n. with two formae, and *Entodinium parvum* forma

monospinosum forma n. from the zebu cattle (*Bos indicus* L., 1758) in Thailand. Jpn. J. Vet. Sci. 45:5, 585-591.

163. Goçmen, B. (1993) Sigir igkembesinde Endosimbiyont Yagayan *Isotricha* Spp. Stein. 1859 (Isotrichidae. Trichostomatida). Jzerine lgik Mikroskobu Duzeyinde MorfoIojik ve SitoIojik Gijzlemler. Tr. J. of Zoolog. 17, 289-301.

154. Ergen, G., Göçmen, B., and Mutaf, B. F. (2000). Ultrastructure of the cortex of the rumen ciliate *Ophryoscolex purkynjei* Stein, 1858 (Entodiniomorphida: Ophryoscolecidae). Turk. J. Zool. 24:4, 385-390.

155. Göçmen, B. (1999). *Ophryoscolex* Stein, 1858 (Protozoa: Ciliophora: Entodiniomorphida) Cinsi Hakk› nda Morfolojik ve Taksonomik Araflt› firmalar. 2, 397-427.

156. Göçmen, B. (2000). New Rumen Ciliates from Turkish Domestic Cattle (*Bos taurus* L.): 2. *Epidinium graini* n. sp.(Ophryoscolecidae, Entodiniomorphida). Turk. J. Zool. 24:1, 23-32.

157. Mishima, T., Katamoto, H., Horii, Y., Kakengi, V. A., and Ito, A. (2009). Rumen ciliates from Tanzanian short horn zebu cattle, Bos taurus indicus, and the infraciliature of Entodinium palmare n. sp. and Enoploplastron stokyi. Eur. J. Protistol. 45:2, 77-86.

158. Göçmen, B. (1999) *Epidinium* Crawley, 1923 (Protozoa: Ciliophora: Entodiniomorphida) Cinsi Hakknda Morfolojik ve Taksonomik Araflt›rmalar. Tr. J. of Zool., 23:2, 831.

159. Göçmen, B., and Öktem, N. (1996). New rumen ciliates from Turkish domestic cattle (*Bos taurus* L.) I. The presence of *Entodinium* dalli Dehority, 1974 with a new forma, *E. dalli* f. *rudidorsospinatum* nf and comparisons with *Entodinium williamsi* n. sp. Eur. J. Prot. 32:4, 513-522.

160. Göçmen B, Akyurtlaklı N, Özbel Y. (2001). İşkembe Siliyatları *Isotricha intestinalis* Stein, 1859 ve *Isotricha prostoma* Stein, 1961 (Trichostomatida: Isotrichidae) Hakkında İnce Yapısal Gözlemler. T. Parazitol. Derg. 25:2, 202-211.

161. Göçmen, B., Tosunoğlu, M., and Mutaf, B. F. (2001). New rumen ciliates from Turkish domestic cattle (*Bos taurus* L.): 3. *Entodinium oektemae* n. sp. and *Entodinium imaii* n. sp.(Entodiniidae, Entodiniomorphida). Turk. J. Zool. 25:3, 269-274.

162. Gürelli, G. (2012). About Ciliates *Entodinium palmare* and *E. okoppensis* (Ciliophora: Ophryoscolecidae) in the Rumen of Domestic Cattles (*Bos taurus taurus*) in the vicinity of Kastamonu. Türk. Paraz. Dergisi. 36:4, 228.

163. Gürelli, G. (2013). *Entodinium dalli m. monospinatum* nm and *Entodinium dalli m. triangulobatum* nm, two new morphotypes of *Entodinium dalli* Dehority, 1974, from Turkish Cattle. Biharean Biol. 7, 69-72.

164. Gürelli, G. (2014). First record of *Diplodinium rangiferi* Dogiel, 1925 (Ophryoscolecidae, Entodiniomorphida) from domestic cattle. Turk. J. Zool. 38:3, 369-372.

165. Gürelli, G. (2014). New rumen ciliate, *Entodinium kastamonicum*, new species (Ophryoscolecidae: Entodiniomorphida) from Turkish domestic cattle, *Bos taurus taurus*. Pakistan J. Zool. 46:1, 93-98.

166. Gürelli, G. (2016). Rumen ciliates of domestic cattle (*Bos taurus taurus*) in Kastamonu, Turkey, with the description of a new species. Eur. J. Protistol. 56, 51-59.

167. Gürelli, G., and Akman, F. T. B. (2017). Rumen ciliate biota of domestic cattle (*Bos taurus taurus*) in İstanbul, Turkey and Infraciliature of *Metadinium medium* (Entodiniomorphida, Ophryoscolecidae). Acta Protozool. 3, 171-180.

168. Köse, S. İ., and Zerek, A. (2018) The first *Buxtonella sulcata* infection in a heifer calf in hatay province. Int. J. Sci. Environ. Technol. 7:5, 1743-1749.

169. Oktem, N., and Goçmen, B. (1996) Tiirkiye Evcil Sigir (*Bos taurus taurus* L.) igkembesinden Yeni Bir Siliyat Grubu (Entodiniomorphida: Ophryoscolecidae) ve Yeni Bir Tiir *Entodinium basoglui* sp. nov. Hakkinda. Tr. J. of Zool. 20271 -278.

170. Correa, O., and Castro, O. (2015). Presencia del protozoario ciliado *Buxtonella sulcata* (Trichostomatia, Balantidiidae) en bovinos en Uruguay. Vet. Montevideo. 51:198, 4-4.

171. Dehority, B. A., Grings, E. E., and Short, R. E. (1999). Effects of Cross-Inoculation From Elk and Feeding Pine Needles On the Protozoan Fauna of Pregnant Cows: Occurrence of Parentodinium Africanum In Domestic US Cattle (*Bos Taurus*). J. Eukaryot. Microbiol. 46:6, 632-636.

172. Rees, C. W. (1930). Studies on the morphology and behaviour of *Buxtonella sulcata* from cattle and of *Balantidium coli* from the pig. Parasitol. 22:3, 314-325.

173. Vasily, D. B., and Mitchell, J. B. (1974). The Identification of Rumen Ciliates from Eastern *Bos taurus*. Trans. Am. Microsc. Soc. 248-253.

174. Wenner, B. A., Wagner, B. K., and Firkins, J. L. (2018). Using video microscopy to improve quantitative estimates of protozoal motility and cell volume. J. Dairy Sci. 101:2, 1060-1073.

175. Becker and Talbot (1927) The protozoan fauna of the rumen and reticulum of amercian cattle. Iowa St. Coll. J. Sci. 1, 345-373.

176. da Silva Barbosa, A., Machado Pereira Bastos, O., M. Antunes Uchôa, C., Verdan Dib, L., and Reis Amendoeira, M. R. (2016). *Balantidium coli* frequency evaluation in pig and nonhuman primate handlers in the state of Rio de Baneiro, Brazil. J. Trop. Pathol. 45:3, 285-293.

177. Bauri, R., Ranjan, R., Deb, A., and Ranjan, R. (2012). Prevalence and sustainable control of *Balantidium coli* infection in pigs of Ranchi, Jahrkahnd, India. Vet.World, 94.

178. Krascheninnikow, S., and Wenrich, D. H. (1958). Some Observations on the Morphology and Division of *Balantidium coli* and *Balantidium caviae*. J. Protozool. 5:3, 196-202.

179. McDonald, J.D. (1922). On *Balantidium coli* (Malmsten) and *Balantidium suis* (sp. nov.) with an account of their neuromotor apparatus. Univ. Calif. Publ. Zool. 20, 243-300.

180. Gürelli, G., and Göçmen, B. (2010). Intestinal ciliate composition found in the feces of the Cypriot wild donkey, *Equus asinus* Linnaeus, 1758. Eur. Protistol. 46:1, 38-42.

181. Olmos, S. E. R. & Ochoterena, E. L. (1968) Protozoarios Ciliados de Mexico XV. Contribucion al conocimiento de algunas especies entozoicas de *Asinus asinus* (Linnaeus). Rev. Soc. Mex. Hist. Nat. 29, 191-208.

182. Kinsella, J. M., Deem, S. L., Blake, S., and Freeman, A. S. (2004). Endoparasites of African forest elephants (*Loxodonta africana cyclotis*) from the Republic of Congo and Central African Republic. Comp. Parasitol. 71:2, 104-111.

183. Timoshenko, O., and Imai, S. (1995). Eleven new species of the genus *Triplumaria* (Entodiniomorphida) from Asian elephant, *Elephas maximus* and African elephant, *Loxodonta africana*. J. Protozool. Res. 5, 157-175.

184. Mandal, D., and Choudhury, A. (1983a). On two new Cycloposthiid ciliates from Indian elephant, *Elephus maximus* L. J. Beng. Natl. Hist. Soc. 2, 13-18.

185. Mandal, D. and Choudhury, A. (1983b). On the new ciliates from Indian elephant. J. Beng. Natl. Hist. Soc. 2, 58.

186. Mandal, D., and Choudhury, A. (1984). A new host record for the ciliate *Polymorphella ampulla* from *Elephas maximus* of Belta, Bihar, India, with a note on its morphology. Giobios New Rep. 3, 103-105.

187. Kofoid, C.A. (1935). On two remarkable ciliate Protozoa from the caecum of the Indian elephant. Proc. Nat. Acad. Sci. 21, 501-06.

188. Ito, A., Honma, H., Gürelli, G., Göcmen, B., Mishima, T., Nakai, Y., Imai, S. (2010). Redescription of *Triplumaria selenica* Latteur et al., 1970 (Ciliophora, Entodiniomorphida) and its phylogenetic position based on the infraciliary bands and 18SSU rRNA gene sequence. Eur. J. Protistol. 46, 180-188.

189. Ito, A., Mishima, T., Nataami, K., Ike, K., Imai, S. (2011). Infraciliature of eight *Triplumaria* species (Ciliophora, Entodiniomorphida) from Asian elephants with the description of six new species. Eur. J. Protistol. 47, 256-273.

190. Obanda, V., Lekolool, I., Kariuki, J., and Gakuya, F. (2007). Composition of intestinal ciliate fauna of free-ranging African elephants in Tsavo West National Park, Kenya. Pachyderm. 42, 92-96.

191. Wolska, M. (1967). Study on the family Blepharocorythidae Hsiung. III. *Raabena bella* gen. n., sp. n. from the intestine of the Indian elephant. Acta protozool. 4, 285-290.

192. Wolska, M. (1968). Study on the family Blepharocorythidae Hsiung. IV. *Pararaabena dentata* gen. n., sp. n. from the intestine of the Indian elephant. Acta Protozool. 5, 219-224.

193. Wolska, M. (1970). *Spirocorys indicus* Wolska, 1969 a ciliate from the intestine of Indian elephant and its systematic position. Acta Protozool. 8, 143-148.

194. Wolska, M. (1986). *Pseudoentodinium elephantis* gen. nov., sp. n. from the Order Entodiniomorphida. Proposition of the New Family Pseudoentodiniidae. Acta Protozool. 25:2, 139-146.

195. Eloff, K. A, Van Hoven W. (1980). Intestinal Protozoa of the African Elephant *Loxodonta africana* (Blumenbach), S. Afr. J. Zool. 15, 83-90.

196. Ito, A., Ishihara, M., and Imai, S. (2014). *Bozasella gracilis* n. sp. (Ciliophora, Entodiniomorphida) from Asian elephant and phylogenetic analysis of entodiniomorphids and vestibuliferids. Europ. J. Protistol. 50:2, 134-152.

197. Gürelli, G. (2016). Intestinal ciliate fauna of the Asian elephant from Gaziantep, Turkey and the description of *Brevitentaculum antebum* ng, n. sp. European journal of protistology, 56, 26-31.

198. Gürelli, G. (2019). New Entodiniomorphid Ciliates, *Buetschlia minuta* n. sp., *B. cirrata* n. sp., *Charonina elephanti* n. sp., from Asian Elephants of Turkey. Zootaxa 4545:3, 419-433.

199. Gürelli, G., and Ito, A. (2014). Intestinal ciliated protozoa of the Asian elephant *Elephas maximus* Linnaeus, 1758 with the description of *Triplumaria izmirae* n. sp. Europ. J. Protistol. 50:1, 25-32.

200. McBee, R. H., Johnson, J. L., and Bryant, M. P. (1969). Ruminal microorganisms from elk. J. Wildl. Manag., 181-186.

201. Grim, J. N., and Clements, K. D. (2013). New Observations on the Ciliate Genus *Vestibulongum* (Pycnotrichidae): Vestibular Ultrastructure, Macronuclear

Endosymbiotic Bacteria, Biogeography, and Evidence for Host Specificity. J. Eukaryot. Microbiol. 60:1, 37-43.

202. Grim, J. N. (1992). Descriptions of Two Sympatric and Phylogenetically Diverse Ciliated Protozoa, *Balantidium zebrascopi* n. sp. and *Paracichlidotherus leeuwenhoeki* n. gen., n. sp., Symbionts in the Intestines of the Surgeonfish, *Zebrasoma scopas*. Trans Am. Microsc. Soc., 149-157.

203. Li, M., Wang, C., Wang, J., Li, A., Gong, X., and Ma, H. (2009). Redescription of *Balantidium polyvacuolum* Li 1963 (Class: Litostomatea) inhabiting the intestines of Xenocyprinae fishes in Hubei, China. Parasitol. Res. 106:1, 177-182.

204. Diamant, A., and Wilbert, N. (1985). *Balantidium sigani* sp. nov., a trichostome ciliate from Red Sea rabbitfish (pisces, siganidae). Arch. Protistenk. 129:1-4, 13-17.

205. Grim, J. N. (1985). *Balantidium prionurium* n. sp., Symbiont in the Intestine of the Surgeonfish, *Prionurus punctatus*. J. Protozool. 32:4, 587-588.

206. Grim, J. N. (1989). The vestibuliferan ciliate *Balantidium acanthuri* n. sp. from two species of the surgeonfish, genus *Acanthurus*. Arch. Protistenk. 137:2, 157-160.

207. Grim, J. N., Clements, K. D., and Byfield, T. (2002). New Species of *Balantidium* and *Pamcichttdotherus* (Ciliophora) Inhabiting the Intestines of Four Surgeonfish Species from the Tuvalu Islands, Pacific Ocean. J. Eukaryot. Microbiol. 49:2, 146-153.

208. Crha, J., Hrabě, V., Koubek, P. Rumen Ciliate Fauna in the Chamois (*Rupicapra rupicapra* L.). Acta. Vet. Brno. 54:3, 141-147.

209. Chen, C. L. (1955). The protozoan parasites from four species of Chinese pond fishes: *Ctenopharyngodon idellus*, *Mylopharyngodon piceus*, *Aristhicthys nobillis* and *Hypophthalmichthys molitrix*. Acta Hydrobiol. Sin.1, 123-164.

210. Li, M., Li, W., Zhang, L., and Wang, C. (2013) *Balantidium honghuensis* n. sp. (Ciliophora: Trichostomatidae) from the rectum of *Rana nigromaculata* and *R. limnocharis* from Honghu Lake, China. Korean J. Parasitol. 51, 427-431.

211. Zhao, W., Li, C., Zhang, D., Wang, R., Zheng, Y., Zou, H., and Li, M. (2018). *Balantidium grimi* n. sp. (Ciliophora, Litostomatea), a new species inhabiting the rectum of the frog *Quasipaa spinosa* from Lishui, China. Parasite, 25.

212. Nie, D. S. (1935) Intestinal ciliates of Amphibia of Nanking. Contr. Biol. Lab. Sci. Soc. China 11:2, 47-95.

213. Jírovec, O. (1930). Über ein neues *Balantidium* aus dem Darmtrakt von *Amblystoma tigrinum*. Parasitol. Res. 3:1, 17-21.

214. Kalavati, C., Narasimhamurti, C. C., and Usharani, Y. (1991). Studies on the endocommensal ciliates of anurans of Andhra Pradesh. Rec. Zool. Surv. India. 141, 1-65.

215. Mahoon, M. S., and Khan, M. I. (1986). Entozoic protozoa of frog *Rana cyanophlyctis* Schneider. Biologia 32, 383-420.

216. Shete, S. G., and Krishnamurthy, R. (1984). Observations on the rectal ciliates of the genus *Balantidium*, Claparede and Lachmann, 1858 from Indian amphibians *Rana tigrina* and *R. cyanophlyctis*. Arch. Protistenk. 128:1-2, 179-194.

217. Bezzenberger, E. (1904). Uber Infusorien aus asiatischlen Anuren. Arch. Protistenk. 3, 138-174.

218. Ray, H. (1932). On the morphology of *Balantidium sushilii* n. sp., from *Rana tigrina* Daud. J. R. Microsc. Soc. 52, 374-382.

219. Rodriguez, J. M. (1939). On the Morphology of *Balantidium kirbyi* n. sp., from the Plathander. J. Parasitol. 25:3, 197.

220. Grim, J. N., and Buonanno, F. (2009). A re-description of the ciliate genus and type species, *Balantidium entozoon*. Europ. J. Protistol. 45:3, 174-182.

221. Bhatia, B. L. (1936). Protozoa: Ciliophora. In: Sewell R. B. S. ed. The fauna of British India, including Ceylon and Burma. London: Taylor & Francis.

222. Dobell, C. C. (1910). On some parasitic protozoa from Ceylon. Spolia Zeylan 7, 65-87.

223. Kuperman, B. I., Matey, V. E., Fisher, R. N., Ervin, E. L., Warburton, M. L., Bakhireva, L., and Lehman, C. A. (2004). Parasites of the African Clawed Frog, *Xenopus laevis*, in Southern California, U.S.A. Comp. Parasitol. 71:2, 229-232.

224. Kornilova, O. A. (2006) Ciliates from the intestine of Yakut horse (Equus caballus). Parazitol. 40:5, 472-8.

225. Wilkinson, R. C., and Van Hoven, W. (1976). Rumen ciliate fauna of the springbok (*Antidorcas marsupialis*) in southern Africa. Afr. Zool. 11:1, 1-22.

226. Gürelli, G., Göçmen, B., and Yildiz, M. Z. (2012). Rumen ciliates from the goitered gazelle (*Gazella subgutturosa*) living in the Şanlıurfa Province of Turkey. Biharean Biol. 6:1, 42-44.

227. Kleynhans, C. J., and Hoven, W. V. (1976). Rumen protozoa of the giraffe with a description of two new species. Afr. J. Ecol. 14:3, 203-214.

228. Carvalho, L. F. P. B., Amorim, G. L., Matos, D. S. D., Batista, Â. M. V., Moraes, A. C. A. D., and Cabral, A. M. D. (2011). Protozoários do rúmen de caprinos submetidos a dieta com casca de soja. Rev. Bras. S. Prod. Anim. 12:1, 244-253.

229. Ebrahimi, S. H., Valizadeh, R., and Miri, V. H. (2018). Rumen Microbial Community of Saanen Goats Adapted to a High-Fiber Diet in the Northeast of Iran. Iranian J. App. Anim. Sci. 8:2.

230. Matsumoto, M., A., Takenaka, T., Kobayashi, and H., Itabashi. (1989). The effects of *Epidinium caudatum* or *Dasytricha ruminantium* on the rumen fermentation and nitrogen metabolism in goats. Asian J. Anim. Sci. 2, 483-484.

231. de la Fuente, G., Belanche, A., Abecia, L., Dehority, B. A., Fondevilla, M. (2009). Rumen protozoal diversity in the *Spanish ibex* (*Capra pyrenaica hispanica*) as compared with domestic goats (*Capra hircus*). Europ. J. Protistol. 42:2, 112-120.

232. Göçmen, B., and Atatür, M. K. (2001). Some Rumen Ciliates (Isotrichidae, Trichostomatida; Epidininae, Ophryoscolecidae) of the Domestic Goat (*Capra hircus* L.) in Turkey. Turk. J. Zool. 26:1, 15-26.

233. Göçmen, B., and Karaglu, A. (2005). Türkiye'nin güneydoğusunda dağılış gösteren evcil keçilerin (*Capra hircus* L.) işkembesinde yaşayan Entodiniid (Entodiniidae, Entodiniomorphida) siliyatlar. Türk. Parazitol. Derg. 29, 211-218.

234. Göçmen, B., and Rastgeldi, S. (2004). A New Rumen Ciliate from the Turkish Domestic Goat (*Capra hircus* L.): *Entodinium salmani* n. sp. (Entodiniidae, Entodiniomorphida). Turk. J. Zool. 28:4, 295-299.

235. Göçmen, B., and Sezgin, Y. (2006). Kuzey Kıbrıs Evcil Keçilerinde (*Capra hircus* L.) Yaşayan İşkembe Siliyatı *Ophryoscolex purkynjei* Stein, 1858 (Sensu Göçmen, 1999). T. Parazitol. Derg. 30:3, 46-51.

236. Göçmen, B., Dehority, B. A., and Rastgeldi, S. (2002). The Recurrence of the Rumen Ciliate *Metadinium banksi* Dehority, 1985 (Ophryoscolecidae, Entodiniomorphida) from Domestic Goats (*Capra hircus* L.) in Southeast Turkey. Turk. J. Zool. 26:4, 367-370.

237. Göçmen, B., Rastgeldi, S., Karaglu, S., and A'Kan, H. K. N. (2005). Rumen ciliated protozoa of the turkish domestic goats (*Capra hircus* L.). Zootaxa, 1091:1, 53-64.

238. Gürelli, G. (2014). Rumen ciliate fauna (Ciliophora, Protista) of Turkish domestic goats living in İzmir, Turkey. Turk. J. Zool. 38:2, 136-143.

239. Mermer, A., Rastgeldi, S., Ergen, G., and Göçmen, B. (2003). Occurence of the rumen ciliate, *Elytroplastron bubali* (Dogiel, 1928) in Turkish domestic goats (*Capra hircus*). T. Parazitol. Derg. 27, 401-403.

240. Rastgeldi, S., and Göçmen, B. (2003). Türkiye Evcil Keçilerinde (*Capra hircus* L.) İşkembe Siliyatları, *Diplodinium crista-galli* ve *Diplodinium flabellum*'un (Entodiniomorphida) Bulunuşu. T. Parazitol. Derg. 27:4, 287-293.

241. Rastgeldi, S., and Göçmen, B. (2003). Turkiye'de dağilis gösteren evcil keçilerin (*Capra hircus* L.) iskembesinde yasayan *Polyplastron multivesiculatum* (Ciliophora: Protista) hakkinda. T. Parazitol. Derg. 27, 71-74.

242. Lubinsky, G. (1964). Ophryoscolecidae of a guanaco from the Winnipeg zoo. Can. J. Zool. 42:1, 159.

243. Alves, L. C., Borges, C. C. A., Silva, S. da, Couto, S. E. R., and Menezes, R. C. (2007). Endoparasitos em cobaias (*Cavia porcellus*) (Mammalia, Rodentia, Caviidae) provenientes de biotérios de criação e experimentação do município do Rio de Janeiro, Brasil. Ciênc. Rural 37:5, 1380-1386.

244. Ito A., Miyazaki, Y., and Imai S. (2002): Descriptions of new *Parentodinium* ciliates in the family Parentodiniidae n. fam. from *Hippopotamus amphibius* in comparison with some entodiniomorphs from horses and cattle. Europ. J.Protist. 37, 405-426.

245. Thurston J. P., and Grain J. (1971). Holotrich ciliates from the stomach of *Hippopotamus amphibius*, with descriptions of two new genera and four new species. J. Protozool.18, 133-141.

246. Thurston, J. P., and Noirot-Timothée, C. (1973). Entodiniomorph ciliates from the stomach of *Hippopotamus amphibius*, with descriptions of two new genera and three new species. J. Protozool. 20, 562-565.

247. Bardele, C. F., Schultheiß, S., Lynn, D. H., Wright, A. D. G., Dominguez-Bello, M. G., and Obispo, N. E. (2017). *Aviisotricha hoazini* n. gen., n. sp., the morphology and molecular phylogeny of an anaerobic ciliate from the crop of the Hoatzin (*Opisthocomus hoazin*), the cow among the birds. Protist, 168:3, 335-351.

248. Carpano, M. (1941). Sopra un nuovo infusorio dell'apparato digerente degli equini *Bertolinella intestinalis* n. g., n. sp. Riv. Parassitol. 5, 45-52.

249. Cedrola, F., Bordim, S., D'Agosto, M., Dias, R. J. P. (2019). Intestinal ciliates (Alveolata, Ciliophora) in Brazilian domestic horses (*Equus caballus* L.) and a review on the ciliate communities associated with horses around the world. Zootaxa 4585, 478-488.

250. Tung, K. -C. (1992). Analysis of the composition and morphology of intestinal ciliates excreted in feces of the riding horses in middle Taiwan. Bull. Facult. Agric. Nat. 41, 53-56.

251. Göçmen, B., Gürelli, G., and Dehority, B. A. (2012). Fecal ciliate composition of Cypriot domestic horses (*Equus caballus* Linnaeus, 1758). Turk. J. Zool. 36:2, 163-170.

252. Gürelli, G., and Göçmen, B. (2010). The occurrence of the hindgut ciliate *Hemiprorodon gymnoposthium* (Ciliophora: Buetschliidae) from domestic horses in Cyprus. Turk. Parazitol. Derg. 34:3, 206-208.

253. Headley, S. A., Kummala, E., and Sukura, A. (2008). *Balantidium coli*-infection in a Finnish horse. Vet. Parasitol. 158:1-2, 129-132.

254. Ike, K., Imai, S., and Ishii, T. (1985). Establishment of intestinal ciliates in newborn horses. Jpn. J. Vet. Sci. 47, 39-43.

255. Ike, K., Nuruki, R., Imai, S., and Ishii, T. (1983a). Composition of intestinal ciliates and bacteria excreted in feces of the racehorse. Jpn. J. Vet. Sci. 45, 157-163.

256. Ike, K., Nuruki, R., Nomoto, Y., Imai, S., and Ishii, T. (1983b). Comparative studies on the intestinal ciliate fauna excreted in the feces of yearlings, blood mares, riding horses and racehorses. Bull. Equine Res. Inst. 20, 63-70.

257. Imai, S., Inami, K., Morita, T., Ike, K., and Ito, A. (1999). Intestinal ciliate composition found in the feces of Japanese native kiso horse. Bull. Nippon Vet. Anim. Sci. Univ. 48, 33-38.

258. Imai, S., Ozeki, K., and Fujita, J. (1979). Scanning electron microscopy of ciliary zones of the ciliate protozoa in the large intestine of the horse. J. Parasitol. 65:3, 434-440.

259. Ito, A., Imai, S., Ogimito, K., and Nakahara, M. (1996). Intestinal ciliates found in the feces of Japanese native Tokara pony, with the description of a new genus and a new species. J. Vet. Med. Sci. 58, 103-108.

260. Kobayashi, Y., Koike, S., Miyaji, M., Hata, H., and Tanaka, K. (2006). Hindgut microbes, fermentation and their seasonal variations in Hokkaido native horses compared to light horses. Ecol. Res. 21:2, 285-291.

261. Ozeki, K., Soichi, Imai, and Katsuno, M. (1973). On the distribution of the ciliated protozoa in the large intestine of horse. Tohoku J. Agric. Res. 24:2, 86-101.

262. Imai, S., and Yamazaki, Y. (1988). Scanning Electron Microscopy of the Adoral Ciliary Zone of *Cycloposthium* Bundle (Ciliophora, Entodiniomorphida). J. Protozool. 35, 578-583.

263. Gürelli, G. Ö. Z. D. E., Canbulat, S. A. V. A. Ş., and Aldayarov, N. (2015). Fecal Ciliate Composition of Domestic Horses (*Equus caballus* Linnaeus, 1758) Living in Kyrgyzstan. Zootaxa 4039:1, 145-156.

264. Güiris, A. D. M., Rojas, H. N. M., Berovides, A. V., Sosa, P. J., Pérez, E. M. E., Cruz, A. E., and Ortega-Pacheco, A. (2010). Biodiversity and distribution of helminths and protozoa in naturally infected horses from the biosphere reserve La Sierra Madre de Chiapas, México. Vet. Parasitol. 170:3-4, 268-277.

265. Chavarria, M. (1933). Estudios protistológicos. II. *Ochoterenaia appendiculata* gen. n., sp. n., nuevo infusorio del intestino del caballo (*Equus caballus* Linn) de México. An. Inst. Biol. Univ. Nac. Aut. Méx. 4, 191-196.

266. Cruces, Rojas, and López-Occotereña, G. E. (1966). Protozoarios ciliados de México XIII. Morfología taxonomía de algunos ciliados entozoicos de *Equus caballus* Linnaeus. Rev. Soc. Mex. Hist. Nat. 27, 49-66.

267. Fernandes, K. A., Kittelmann, S., Rogers, C. W., Gee, E. K., Bolwell, C. F., Bermingham, E. N., and Thomas, D. G. (2014). Faecal Microbiota of Forage-Fed Horses in New Zealand and the Population Dynamics of Microbial Communities following Dietary Change. PLoS ONE, 9:11, e112846.

268. Strüder-Kypke, M. C., Kornilova, O. A., and Lynn, D. H. (2007). Phylogeny of trichostome ciliates (Ciliophora, Litostomatea) endosymbiotic in the Yakut horse (*Equus caballus*). Europ. J. Protistol. 43:4, 319-328.

269. Gürelli, G., and Göçmen, B. (2009). İzmir civarındaki evcil atların (*Equus caballus* Linnaeus, 1758) arka bağırsağında tespit edilen siliyatlardan *Blepharocorys curvigula* (Ciliophora: Blepharocorythidae) hakkında. Turk. Parazitol. Derg. 33, 169-171.

270. Gürelli, G., and Göçmen, B. (2009). The occurrence of the hindgut ciliate *Blepharocorys curvigula* (Ciliophora: Blepharocorythidae) from horses (*Equus caballus* Linnaeus, 1758) in the vicinity of Izmir. Turk. Parazitol. Derg. 33:2, 169-171.

271. Gürelli, G., and Göçmen, B. (2011). Intestinal ciliate composition found in the feces of the Turk rahvan horse *Equus caballus*, Linnaeus 1758. Europ. J. Protistol. 47:4, 245-255.

272. Gürelli, G., and Göçmen, B. (2012). Intestinal ciliate composition found in the feces of racing horses from Izmir, Turkey. Europ. J. Protistol. 48:3, 215-226.

273. Gürelli, G., and Göçmen, B. (2012). Occurence of the hindgut ciliates *Paraisotricha colpoidea* and *P. minuta* (Ciliophora: Paraisotrichidae) in horses in Turkey. Türk. Parazitol. Derg. 36:1, 28.

274. Gürelli, G., and Göçmen, B. (2012). Ülkemiz Atlarında (*Equus caballus*) Linnaeus, 1758 Yaşayan Arka Bağırsak Siliyatları *Parasiotricha colpoidea* Fiorentini, 1890 ve *P. minuta* Hsiung, 1930 (Ciliophora: Paraisotrichidae) Hakkında. Turk. Parazitol. Derg. 36, 28-32.

275. Gürelli, G., and Göçmen, B. (2014). Morphological Features of *Bundleia* spp. (Ciliophora: Entodiniomorphida: Buetschliidae) and Ontogenesis in *Bundleia* Cunha and Muniz 1928. Türk. Parazitol. Derg. 38:4, 239.

276. Strelkow, A. (1928). Nouvelles especes du genre *Cycloposthium* habitant l'intestin du cheval. Ann. Parasitol. Hum. Comp. 6, 164-178.

277. Strelkow, A. (1939). Parasitical infusoria from the intestine of Ungulata belonging to the family Equidae. Urchen Zap. Uchenye Zapiski. Leningrad Pedagog. Inst. Gertsena, 17, 262-374.

278. Gassovsky, G. (1919). Notes et communications. On the microfauna of the intestine of the horse. Trav. Soc. Nat. Petrograd. 49, 20-37.

279. Laho, T., Váradyová, Z., Mihaliková, K., and Kišidayová, S. (2013). Fermentation Capacity of Fecal Microbial Inocula of Przewalski Horse, Kulan, and Chapman Zebra and Polysaccharide Hydrolytic Activities of Fecal Microbial Constituents (Ciliates and Bacteria) of Kulan and Chapman Zebra. J. Equine Vet. Sci. 33:3, 143-149.

280. Hsiung, T. -S. (1929). On *Didesmis spiralis* sp. nov., a new Ciliate from the large intestine of the horse. Trans. Amer. Mic. Soc. 48, 209-213.

281. Hsiung, T. -S. (1930). A monograph on the protozoa of the large intestine of the horse. Iowa State Coll. J. Sci. 4, 359-423.

282. Hsiung, T.-S. (1935a). Notes on the known species of *Triadinium* with the description of a new species. Bull. Fan. Mem. Inst. Biol. 6, 21-32.

283. Hsiung, T. -S. (1935b). On some new species from the mule, with the description of a new genus. Bull. Fan. Mem. Inst. Biol. 6, 81-94.

284. Hsiung, T. -S. (1936). A survey of the ciliates of Chinese equines. Bull. Fan. Mem. Inst. Biol. 6, 289-304.

285. Wertheim, P. (1935b). Infuorien aus dem Widerkauermagen von Gebiete Jugoslawiens nebst einer Ubersicht dieser Tierchen von Balkanhalbinsel Bereich und ein kurzer Bericht tiber die Pferdedarm infusorien, zugleich Revision der Familie Ophryoscolecidae. Vet. Arch. 5, 386-536.

286. Al-Hasan A., Ali A., Al-Hasan A., Rakib F. K., Alam M. A., and Mondal M. M. (2015). Prevalence of *Balantidium coli* infection in man in Mymensingh, Bangladesh. Int. J. Nat. Soc. Sci. 2, 33-36.

287. Cox F. E. (2005). Human balantidiasis in Iran: are camels' reservoir hosts. Tred. Parasitol. 21, 553.

288. Imai, S., Kudo, H., Fukuta, K., Abudullah, N., Ho, Y. W. and Onodere, R. (1995). *Isotricha jalaludinii* n. sp. Found from the rumen of lesser mouse deer, *Tragulus javanicus*, in Malaysia. J. Eukaryotic Microbiol, 42:75-77.

289. Cerón Cucchi, M., Marcoppido, G., Morici, G., Dekker, A., De La Fuente, G., Fondevila, M., and Cravero, S. (2016). Ciliate protozoa of the forestomach of llamas (*Lama glama*) from locations at different altitude in Argentina. Zootaxa. 20, 49-56.

290. Cameron S. L. and O'Donoghue P. J. and Adlard R. D. (2000b): Novel isotrichid ciliates endosymbiotic in Australian macropodid marsupials. Syst. Parasitol. 46, 45-57.

291. Cameron S. L., O'Donoghue P. J. and Adlard R. D. (2001a): Four new species of *Macropodinium* (Ciliophora: Litostomatea) from Australian wallabies and pademelons. J. Euk. Microbiol. 48, 542-555.

292. Cameron, S. L., and O'Donoghue, P. J. (2002). The ultrastructure of *Amylovorax dehorityi* comb. nov. and erection of the Amylovoracidae fam. nov. (Ciliophora: Trichostomatia). Eur. J. Protistol. 38:1, 29-44.

293. Cameron, S. L., and O'Donoghue, P. J. (2002). Trichostome ciliates from Australian marsupials. I. *Bandia* gen. nov. (Litostomatea: Amylovoracidae). Eur. J. Protistol. 38:4, 405-429.

294. Cameron, S. L., and O'Donoghue, P. J. (2003). Trichostome ciliates from Australian marsupials. II. *Polycosta* gen. nov. (Litostomatea: Polycostidae fam. nov.). Eur. J. Protistol. 39:1, 83-99.

295. Cameron, S. L., and O'Donoghue, P. J. (2001). Stomatogenesis in the ciliate genus *Macropodinium* Dehority, 1996 (Litostomatea: Macropodiniidae). Eur. J. Protistol. 37:2, 199-206.

296. Cameron, S. L., and O'Donoghue, P. J. (2003). Trichostome ciliates from Australian marsupials. III. *Megavestibulum* gen. nov.(Litostomatea: Macropodiniidae). Eur. J. Protistol. 39:2, 123-137.

297. Cameron, S. L., and O'Donoghue, P. J. (2003). Trichostome ciliates from Australian marsupials. IV. Distribution of the ciliate fauna. European journal of protistology, 39(2), 139-147.

298. Cameron, S., O-Donoghue, P. and Adlard, R. (2000). First record of *Cycloposthium edentatum* Strelkow, 1928 from the black stripped wallaby, *Macropus dorsalis*. Parasitol. Res. 86, 158-162.

299. Dehority, B. A. (1996). A New Family of Entodiniomorp Protozoa from the Marsupial Forestomach, with Descriptions of a New Genus and Five New Species. J. Eukaryot. Microbiol. 43:4, 285-295.

300. Derlet, R. W., and Carlson, J. R. (2002). An Analysis of Human Pathogens Found in Horse/Mule Manure Along the John Muir Trail in Kings Canyon and Sequoia and Yosemite National Parks. Wilderness Environ. Med. 13:2, 113-118.

301. Lubinsky, G. (1963). *Metadinium caudatum* sp. n. - A rumen ciliate of Muskox from Northern Canada. Can. J. Zool. 41:1, 29-32.

302. Dehority, B. A. (1985). Rumen Ciliates of Musk- Oxen (*Ovibos moschatus* Z.) from the Canadian Arctic 1. J. Protozool. 32:2, 246-250.

303. O'Donoghue, P. J., Gasser, R. B., and Tribe, A. (1993). New host record for the entodiniomorphid ciliate, *Troglodytella abrassarti*, from siamangs (*Hylobates syndactylus*). J. Protozool. 23:3, 415-418.

304. Pomajbíková, K., Petrželková, K. J., Profousová, I., Petrášová, J., Kišidayová, S., Varádyová, Z., and Modrý, D. (2009). A survey of entodiniomorphid ciliates in chimpanzees and bonobos. Am. J. Phys. Anthropol. 142, 42-48.

305. Da Silva Barbosa, A., Pissinatti, A., Dib, L. V., de Siqueira, M. P., Cardozo, M. L., Fonseca, A. B. M., Amendoeira, M. R. R. (2014). *Balantidium coli* and other gastrointestinal parasites in captives non-human primates of the Rio de Janeiro, Brazil. J. Med. Primatol. 44:1, 18-26.

306. Drakulovski, P., Bertout, S., Locatelli, S., Butel, C., Pion, S., Mpoudi-Ngole, E., and Mallié, M. (2014). Assessment of gastrointestinal parasites in wild chimpanzees (*Pan troglodytes troglodytes*) in southeast Cameroon. Parasitol. res. 113:7, 2541-2550.

307. Ibrahim, H., Mamadou, B. K., Martine, P., Eric, D., Didier, R. and Fadi, B. (2014) Pathogenic Eukaryotes in Gut Microbiota of Western Lowland Gorillas as Revealed by Molecular Survey. Sci. Rep. 6417, 4.

308. Freeman, A. S., Kinsella, J. M., Cipolletta, C., Deem, S. L., and Karesh, W. B. (2004). Endoparasites of Western Lowland Gorillas (*Gorilla gorilla gorilla*) at Bai Hokou, Central African Republic. J. Wildl. Dis. 40:4, 775-781.

309. Vlčková, K., Pafčo, B., Petrželková, K. J., Modrý, D., Todd, A., Yeoman, C. J., and Nelson, K. E. (2018). Relationships between gastrointestinal parasite infections and the fecal microbiome in free-ranging Western Lowland Gorillas. Front. Microbiol. 15:9, 1202.

310. Li, H. L., Li, Q., Dong, L., Li, J., Zou, F. C., Zhang, L. (2014). Prevalence of *Balantidium coli* Infection in Bred Rhesus Monkeys (*Macaca mulatta*) in Guangxi, southern China. Iran. J. Parasitol. 9, 125-128.

311. Modrý, D., Petrželková, K. J., Pomajbíková, K., Tokiwa, T., Křížek, J., Imai, S., and Šlapeta, J. (2009). The occurrence and ape- to- ape transmission of the entodiniomorphid ciliate *Troglodytella abrassarti* in captive gorillas. J. Eukaryot. Microbiol. 56:1, 83-87.

312. Petrželková, K. J., Schovancová, K., Profousová, I., Kišidayová, S., Váradyová, Z., Pekár, S and Modrý, D. (2012). The Effect of Low- and High- Fiber Diets on the Population of Entodiniomorphid Ciliates *Troglodytella abrassarti* in Captive Chimpanzees (*Pan troglodytes*). Am. J. Primatol. 74:7, 669-675.

313. Profousová, I., Petrželková, K. J., Pomajbíková, K., and Modrý, D. (2011). Survival and Morphologic Changes of Entodiniomorphid Ciliate *Troglodytella abrassarti* in Chimpanzee Feces. J. Zoo. Wildl. Med. 42:1, 69-74.

314. Hasegawa, H., Kano, T., and Mulavwa, M. (1983). A parasitological survey on the feces of pygmy chimpanzees, *Pan paniscus*, at Wamba, Zaire. Primates. 24:3, 419-423.

315. Brumpt, E. and Joyeux, Ch. (1912) Sur un infusoire nouveau parasite du chimpanzé *Troglodytella abrassarti* n. g. n. sp. Bull. Soc. Path. exot. 5, 499-503.

316. Goussard, B., Collet, J. Y., Garin, Y., Tutin, C. E., and Fernandez, M. (1983). The intestinal entodiniomorph ciliates of wild lowland gorillas (*Gorilla gorilla gorilla*) in Gabon, West Africa. J. Med. Primatol. 12:5, 239-249.

317. Imai, S., Ikeda, S. I., Collet, J. Y., and Bonhomme, A. (1991). Entodiniomorphid ciliates from the wild lowland gorilla with the description of a new genus and three new species. Eur. J. Protistol. 2:3/4, 270-278.

318. Landsoud-Soukate, J., Tutin, C. E. G., and Fernandez, M. (1995). Intestinal parasites of sympatric gorillas and chimpanzees in the Lopé Reserve, Gabon. Ann. Trop. Med. Parasitol. 89:1, 73-79.

319. Cunha, A., and Muniz., J. (1930). Do phenomeno de endomixis em ciliados do gênero *Balantidium*. Mem. Inst. Oswaldo Cruz. 8, 5.

320. Sá, R. M., Petrášová, J., Pomajbíková, K., Profousová, I., Petrželková, K. J., Sousa, C., Modrý, D. (2013). Gastrointestinal symbionts of chimpanzees in Cantanhez National Park, guinea-bissau with respect to habitat fragmentation. Am. J. Primatol. 75:10, 1032-1041.

321. Mul, I. F., Paembonan, W., Singleton, I., Wich, S. A., and van Bolhuis, H. G. (2007). Intestinal parasites of free-ranging, semicaptive, and captive *Pongo abelii* in Sumatra, Indonesia. Int. J. Primatol. 28:2, 407-420.

322. Nakauchi, K. (1990) Detection of Balantidium coli from evacuated feces in cynomolgus monkeys (*Macaca fascicularis*). Jpn. J. Vet. Res. 52:6, 1323-1324.

323. Nakauchi, K. (1999) The prevalence of *Balantidium coli* infection in fifty-six mammalian species. J. Vet. Sci. 61:1, 63-65.

324. Ito, A., Eckardt, W., Stoinski, T. S., Gillespie, T. R., and Tokiwa, T. (2016). Prototapirella ciliates from wild habituated Virunga mountain gorillas (*Gorilla beringei beringei*) in Rwanda with the descriptions of two new species. Eur. J. Protistol. 54, 47-58.

325. Ito, A., Eckardt, W., Stoinski, T. S., Gillespie, T. R., and Tokiwa, T. (2017). *Gorilloflasca africana* ng, n. sp. (Entodiniomorphida) from wild habituated Virunga mountain gorillas (*Gorilla beringei beringei*) in Rwanda. Eur. J. Protistol. 60, 68-75.

326. Ito, A., Eckardt, W., Stoinski, T. S., Gillespie, T. R., and Tokiwa, T. (2018). Three new *Troglodytella* and a new *Gorilloflasca* ciliates (Entodiniomorphida) from mountain gorillas (*Gorilla beringei beringei*) in Rwanda. Eur. J. Protistol. 65, 42-56.

327. Howells, M. E., Pruetz, J., and Gillespie, T. R. (2010). Patterns of gastro-intestinal parasites and commensals as an index of population and ecosystem health: the case of

sympatric western chimpanzees (*Pan troglodytes verus*) and guinea baboons (*Papio hamadryas papio*) at Fongoli, Senegal. Am. J. Primatol. 73:2, 173-179.

328. Irbis, C., Garriga, R., Kabasawa, A., and Ushida, K. (2008). Phylogenetic analysis of *Troglodytella abrassarti* isolated from Chimpanzees (*Pan troglodytes verus*) in the wild and in captivity. J. Gen. Appl. Microbiol. 54:6, 409-413.

329. Gillespie, T. R., Lonsdorf, E. V., Canfield, E. P., Meyer, D. J., Nadler, Y., Raphael, J., Travis, D. A. (2010). Demographic and ecological effects on patterns of parasitism in eastern chimpanzees (*Pan troglodytes schweinfurthii*) in Gombe National Park, Tanzania. Am. J. Phys. Anthropol. 143:4, 534-544.

330. Kalousová, B., Piel, A. K., Pomajbíková, K., Modrý, D., Stewart, F. A., and Petrželková, K. J. (2014). Gastrointestinal parasites of savanna chimpanzees (*Pan troglodytes schweinfurthii*) in Ugalla, Tanzania. Int. J. Primatol. 35:2, 463-475.

331. Kaur, T., Singh, J., & Lindsay, D. S. (2010). Prevalence of *Troglodytella abrassarti* Brumpt and Joyeux, 1912 in Wild Chimpanzees (*Pan troglodytes schweinfurthii*) at Mahale Mountains National Park in Western Tanzania. J. Parasitol. 96:1, 209-211.

332. Trabalza, M. M., Capecci, A., Riganelli, N., Acuti, G., Antonini, C., Olivieri, O. (2005) Dietary preferences and ruminal protozoal populations in roe deer (*Capreolus capreolus*), fallow deer (*Dama dama*) and mouflon (*Ovis musimon*). Ital. J. Anim. Sci. 4: 2, 401-403.

333. McGrew, W. C., Tutin, C. E. G., Collins, D. A., and File, S. K. (1989). Intestinal parasites of sympatric *Pan troglodytes* and *Papio* spp. at two sites: Gombe (Tanzania) and Mt. Assirik (Senegal). Am. J. Primatol. 17:2, 147-155.

334. Murray, S., Stem, C., Boudreau, B., and Goodall, J. (2000). Intestinal parasites of baboons (*Papio cynocephalus anubis*) and chimpanzees (*Pan troglodytes*) in Gombe National Park. J. Zoo. Wildl. Med. 176-178.

335. Petrželková, K. J., Hasegawa, H., Appleton, C. C., Huffman, M. A., Archer, C. E., Moscovice, L. R., and Kaur, T. (2010). Gastrointestinal parasites of the chimpanzee population introduced onto Rubondo Island National Park, Tanzania. Am. J. Primatol. 72:4, 307-316.

336. Ashford, R. W., Lawson, H., Butynski, T. M., and Reid, G. D. F. (1996). Patterns of intestinal parasitism in the mountain gorilla *Gorilla gorillain* the Bwindi-Impenetrable Forest, Uganda. J. Zool. 239:3, 507-514.

337. Ashford, R. W., Reid, G. D. F., and Wrangham, R. W. (2000). Intestinal parasites of the chimpanzee *Pan troglodytes* in Kibale Forest, Uganda. Ann. Trop. Med. Parasitol. 94:2, 173-179.

338. Muehlenbein, M. P. (2005). Parasitological analyses of the male chimpanzees (*Pan troglodytes schweinfurthii*) at Ngogo, Kibale National Park, Uganda. Am. J. Primatol. 65:2, 167-179.

339. Tokiwa, T., Modrý, D., Ito, A., Pomajbíková, K., Petrželková, K. J., and Imai, S. (2010). A new entodiniomorphid ciliate, *Troglocorys cava* ng, n. sp., from the wild eastern chimpanzee (*Pan troglodytes schweinfurthii*) from Uganda. J. Eukaryot. Microbiol. 57:2, 115-120.

340. Lee, R. V., Prowten, A. W., Anthone, S., Satchidanand, S. K., Fisher, J. E., and Anthone, R. (1990). Typhlitis Due to *Balantidium coli* in Captive Lowland Gorillas. Clin. Infect. Dis. 12:6, 1052-1059.

341. Lubinsky, G. (1958). Ophryoscolecidae (Ciliata: Entodiniomorphida) of the reindeer (*Rangifer tarandus* L.) from the Canadian arctic: II. *Diplodiniinae*. Can. J. Zool.. 36:6, 937-959.

342. Imai, S., Oku, Y., Morita, T., and Ike, K. (2004). Rumen ciliate protozoal fauna of reindeer in Inner Mongolia, China. J. Vet. Sci. 66:2, 209-212.

343. Hoare, C.A. (1937) A new cycloposthiid ciliate (*Triplumaris hamertoni* gen. n., sp. n.), parasitic in the Indian rinocheros. Parasitol. 29, 559-569.

344. Buisson, J. (1923) Sur quelques Infusoires nouveaux ou peu connus parasites des Mammiferes. Ann. Parasitol. Hum. Comp. 1, 209-246.

345. Obanda, V., Gakuya, F., Lekolool, I., Chege, S., Okita, B., and Manyibe, T. (2008). Ciliated intestinal protozoa of black (*Diceros bicornis michaeli*) and white rhinoceroses (*Ceratotherium simum simum*) in Kenya. Afr. J. Ecol. 46:2, 144-148.

346. Gilchrist, F. M. C., Van Hoven, W., and Stenson, M. O. (1994). Five new species of Trichostomatida (ciliated protozoa) from the colon of wild African rhinoceroses. Syst. Parasitol. 28:3, 187-196.

347. Van Hoven, W., Gilchrist, F. M. C., Liebenberg, H., and Van Der Merwe, C. F. (1998). Three new species of ciliated protozoa from the hindgut of both white and black wild African rhinoceroses. Onderstepoort J. Vet. Res., 65, 87-95.

348. Ito, A., Van Hoven, W., Miyazaki, Y., and Imai, S. (2006). New entodiniomorphid ciliates from the intestine of the wild African white rhinoceros belong to a new family, the Gilchristidae. Eur. J. Protistol. 42:4, 297-307.

349. Ito, A., Van Hoven, W., Miyazaki, Y., and Imai, S. (2008). Two new entodiniomorphid *Triplumaria* ciliates from the intestine of the wild African white rhinoceros. Eur. J. Protistol. 44:2, 149-158.

350. Van Hoven W., Gilchrist F. and Hamilton-Attwell V. (1987). Intestinal ciliated protozoa of African rhinoceros: Two new genera and five new species from the white rhino (*Ceratotherium simum* Burchell, 1817). J. Protozool. 34, 338-342.

351. Van Hoven, W., Gilchrist, F. M. C., and Hamilton- Attwell, V. L. (1988). A new family, genus, and seven new species of Entodiniomorphida (Protozoa) from the gut of African rhinoceros. J. Protozool. 35:1, 92-97.

352. Li, M., Wang, J., Zhang, J., Gu, Z., Ling, F., Ke, X., and Gong, X. (2007). First report of two *Balantidium* species from the Chinese giant salamander, Andrias davidianus: *Balantidium sinensis* Nie 1935 and *Balantidium andianusis* n. sp.. J. Parasitol. Res. 102:4, 605-611.

353. Pal N. L., Dasgupta, B. (1978) Observations on 2 new species of *Balantidium* in the Indian Salamander *Tylototriton verrucosus caudata* Salamandridae. Proc. Zool. Soc. (Calcutta). 31:1/2, 47-52.

354. Fernández Galiano, D. (1951). *Balantidium galianoi*, una nueva especie parásita del gallipato (*Molge* (*Pleurodeles*) *waltlii*) Michah.). Trab. Inst. Cienc. nat. "José de Acosta". 3, 115.

355. Cedrola, F., Martinele, I., Rossi, M., de Medeiros, G. R., de Arruda Santos, G. R., de Carvalho, F. F. R., and d'Agosto, M. (2013). Protozoários ciliados do rúmen de ovinos do nordeste brasileiro e infraciliatura de *Enoploplastron triloricatum* (Dogiel, 1925). Principia. 17, 71-79.

356. Cedrola, F., Martinele, I., Dias, R. J. P., Fregulia, P., and D'agosto, M. (2016). Rumen ciliates in Brazilian sheep (*Ovis aries*), with new records and redescription of *Entodinium contractum* (Entodiniomorphida: Ophryoscolecidae). Zootaxa. 4088:2, 292-300.

357. Cedrola, F., Dias, R. J. P., Martinele, I., and D'agosto, M. (2017). Description of *Diploplastron dehorityi* sp. nov. (Entodiniomorphida, Ophryoscolecidae), a new rumen ciliate from Brazilian sheep (*Ovis aries*). Zootaxa. 4258:6, 581-585.

358. Cedrola, F., Dias, R. J. P., Martinele, I., and D'Agosto, M. (2017). Polymorphism and inconsistencies in the taxonomy of Diplodinium anisacanthum da Cunha, 1914 (Ciliophora, Entodiniomorphida, Ophryoscolecidae) and taxonomic notes on the genus Diplodinium Schuberg, 1888. Zootaxa 4306:2, 249-260.

359. Cedrola, F., Rossi, M. F., Martinele, I., D'Agosto, M. and Dias, R. J. P. (2018). Morphology and description of infraciliary bands pattern in four *Metadinium* Awerinzew & Mutafowa, 1914 species (Ciliophora, Entodiniomorphida, Ophryoscolecidae) with taxonomic notes on the genus. Zootaxa. 4500:4, 574-580.

360. de Matos, D. S., Guim, A., Batista, Â., dos Santos, M. V. F., Correa, I. M., Santos, G. D. A., and Lopes, C. D. A. (2008). Rumen ciliate protozoa in sheep raising in the

caatinga region of Pernambuco State, Brazil. Rev. Bras. Saude e Prod. Anim. 9:2, 270-279.

361. Freitas, C. E. S., Duarte, E. R., Alves, D. D., Martinele, I., D'Agosto, M., Cedrola, F., and Beltran, M. (2017). Sheep fed with banana leaf hay reduce ruminal protozoa population. Trop. Anim. Health Prod. 49:4, 807-812.

362. Martinele, I., and D'agosto, M. (2007) Variação estacional das populações de protozoários ciliados (Protista, Ciliophora) do rúmen de ovinos (*Ovis aries* L.) mantidos em pastagens naturais no semi-árido de Pernambuco, Brasil. Rev. Bras. Zooc. 9, 238.

363. Martinele, I ; Santos, G. R. A. ; Matos, D.S. ; Batista, A.M.V. ; D'Agosto, M. (2008) Protozoários ciliados do rúmen de ovinos mestiços Santa Inês mantidos em pastagem natural de caatinga. Rev. Bras. Saúde Prod. Anim. 9, 280-292.

364. Martinele, I ; Silva, L. F., Agosto, M., Muniz, E. N., SÁ, J. L., Santos, G. R. A. (2014) Abundance and diversity of rumen protozoa in lambs fed *Gliricidia sepium* silage. Rev. Bras. Zoot. 43, 436-439.

365. Martinele, I., Santos, G.R.A., Matos, D.S., Batista, A.M.V., D'Agosto, M. (2010) Diet botanical composition and rumen protozoa of sheep in Brazilian Semi-Arid area. Arch. Zootec; 59, 226.

366. Martinele, I., and D'Agosto, M. (2008). Predation and cannibalism among ciliate protozoans (Ciliophora: Entodiniomorphida: Ophryoscolecidae) in the rumen of sheep (*Ovis aries*). Rev. Brasil. Zool. 25:3, 451-455.

367. Matos, D. S. D., Guim, A., Batista, Â. M. V., Santos, M. V. F. D., Correa, I. M., Santos, G. R. D. A., and Lopes, C. R. D. A. (2008). População de protozoários ciliados no rúmen de ovinos criados na caatinga de Pernambuco. Rev. Bras. Saúde Prod. Anim. 9, 2.

368. Neto, S., Adelson, J., Martinele, I., Cedrola, F., Santos, G. R. D. A., Muniz, E. N., and D'AGOSTO, M. (2017). Apparent digestibility and rumen protozoal profile of sheep fed cassava wastewater. Rev. Bras. Saúde Prod. Anim. 18:2, 327-336.

369. Godfrey, S. I., Nagaraja, T. G., Winslow, S. W. and Rowe, J. B. (1995). Rumen microbial adaptation to long-term feeding of virginiamycin in sheep fed barley and virginiamycin as a supplement. Aust. J. Agric. Res, 46:6, 1149.

370. Ivan, M., Neill, L., Forster, R., Alimon, R., Rode, L. M., and Entz, T. (2000). Effects of *Isotricha*, *Dasytricha*, *Entodinium*, and total fauna on ruminal fermentation and duodenal flow in wethers fed different diets. J. Dairy. Sci. 83:4, 776-787.

371. Wright, A. D. G., and Lynn, D. H. (1997). Phylogenetic analysis of the rumen ciliate family Ophryoscolecidae based on 18S ribosomal RNA sequences, with new sequences from *Diplodinium*, *Eudiplodinium*, and *Ophryoscolex*. Can. J. Zool. 75:6, 963-970.

372. Wright, A.-D. G., Dehority, B. A., and Lynn, D. H. (1997). Phylogeny of the Rumen Ciliates *Entodinium*, *Epidinium* and *Polyplastron* (Litostomatea: Entodiniomorphida) Inferred from Small Subunit Ribosomal RNA Sequences. J. Eukaryot. Microbiol. 44:1, 61-67.

373. Huang, J., and Li, Y. (2018). Rumen methanogen and protozoal communities of Tibetan sheep and Gansu Alpine Finewool sheep grazing on the Qinghai–Tibetan Plateau, China. BMC microbiol. 18:1, 212.

374. Göçmen, B., and Gürelli, G. (2009). Rumen Entodiniid ciliated protozoan fauna (Entodiniomorphida: Entodiniidae) of domestic sheep (*Ovis ammon aries* L.) from Northern Cyprus, with a description of a new species, *Entodinium cypriensis* sp. nov. Turk. J. Zool. 33:2, 169-180.

375. Göçmen, B., and Gürelli, G. (2009). The occurence of the rumen ciliate *Entodinium constrictum* Dehority, 1974 (Entodiniidae, Entodiniomorphida) from domestic sheep (*Ovis ammon aries* L.) in Northern Cyprus. North-West. J. Zool., 5:2, 301-306.

376. Göçmen, B. (2001) The rumen ciliate fauna of domestic sheep (*Ovis ammon aires*) from the Turkish Republic of Northern Cyprus. J. Eukaryot. Microbiol. 48:4, 455-459.

377. Furness, D. N., and Butler, R. D. (1985). The Cytology of sheep rumen ciliates. II. Ultrastructure of *Eudiplodinium maggii*. J. Protozool. 32:1, 205-214.

378. Furness, D. N., and Butler, R. D. (1985). The Cytology of sheep rumen ciliates. III. Ultrastructure of the genus (Stein). J. Protozool. 32:4, 699-707.

379. Chalechale, A., Karimi, I., Hadipour, M., and Ortiz-Maya, J. (2011). Rumen ciliate fauna of Sanjabi sheep: the first taxonomic report of Iran complemented with a mini-review in Middle East. Global Vet. 7:2, 100-107.

380. Ebrahimi, S. H., Valizadeh, R., Miri, V. H., and Janssen, P. H. (2017). A study of rumen microbial community of Baluchi lambs fed a high concentrate diet containing conventional ingredients. Adv. Res. Microb. Met. Tech.

381. Karimizadeh, E., Chaji, M., and Mohammadabadi, T. (2017). Effects of physical form of diet on nutrient digestibility, rumen fermentation, rumination, growth performance and protozoa population of finishing lambs. Anim. Nut. 3:2, 139-144.

382. Imai, S., and Ogimoto, K. (1978). Scanning electron and fluorescent microscopic studies on the attachment of spherical bacteria to ciliate Protozoa in the ovine rumen. Nihon juigaku zasshi. Jpn. J. Vet. Sci. 40:1, 9-19.

383. Purevtsogt, D., Zolzaya, M., Shirchin, D., and Dugersuren, J. (2016). Some results of study on counts and morphology of rumen ciliate protozoa in pasture-raised Mongolian sheep lamb. Mongolian J. Agric. Sci. 19:3, 16-21.

384. Lubinsky, G. (1957a). Studies on the evolution of Ophryoscolecidae (Ciliata: Oligotricha). I. A new species of *Entodinium* with 'caudatum', 'loboso-spinosum' and 'dubardi' forms and some evolutionary trends in the genus *Entodinium*. Can. J. Zool. 35, 111-133.

385. Zeitz, J. O., Amelchanka, S. L., Michałowski, T., Wereszka, K., Meile, L., Hartnack, S., and Soliva, C. R. (2012). Effect of the rumen ciliates *Entodinium caudatum*, *Epidinium ecaudatum* and *Eudiplodinium maggii*, and combinations thereof, on ruminal fermentation and total tract digestion in sheep. Arch. Anim. Nut. 66:3, 180-199.

386. Bush, M., and Kofoid, C. A. (1948). Ciliates from the Sierra Nevada Bighorn*, Ovis Canadensis* Sierrae Grinnell. University of California Press.

387. Göçmen, B. (2003). Kıbrıs Evcil Koyunlarında Yaşayan Ophryoscolecid (Entodiniomorphida) İşkembe Siliyat Cinsi, *Epidinium* Crawley, 1923. Türk. Parazitol. Derg. 27:4, 280-286.

388. Göçmen, B., Torun, S., and Öktem, N. (1999). Türkiye Evcil Koyun (*Ovis ammon aries*)'lar› n› n‹ flkembe Siliyat (Protozoa: Ciliophora) Faunas› Hakk› nda Bir Ön Çal› flma: II-Familya Ophryoscolecidae (Entodiniomorphida). J. Zool. 23:473-490.

389. Gürelli, G. (2017). Rumen Ciliate Fauna of Domestic Sheep (*Ovis aries*) in İzmir, Turkey and Scanning Electron Microscopic Observations. Zootaxa 4286:4, 545-554.

390. Göçmen, B., Torun, S., and Öktem, N. (1999). A Preliminary Study on the Rumen Ciliate Fauna of Turkish Domestic Sheep (*Ovis ammon aries*): II-Family Ophryoscolecidae (Entodiniomorphida). Turk. J. Zool. 23, 473-490.

391. Torun, N., Okten, S., Göçmen, B., Ege, B. Ilniversitesi Fen Fakuitesi Biyoloji. Turkiye Evcil Koyun (*Ovis ammon aries*)'larlnln lqkembe Siliyat (Protozoa: Ciliophora) Faunas1 Hakkinda Bir on Caliqma: I-Familya Isotrichidae (Trichostomatida) ve Entodiniidae (Entodiniomorphida). Turk. J. Zool. 21, 475-502.

392. Whitenton, R. O. (1927). Ciliates of the Stomach of the Sheep. Proceedings of the Oklahoma Academy of Science 7, 43-46.

393. Alvarado Palacios, M. C. R. (2018). Presencia de parásitos gastrointestinales en la danta centroamericana (*Tapirus bairdii*) y la relación de la composición de parásitos en sus letrinas con variables ambientales en la región noroeste de la Cordillera de Talamanca, Costa Rica.

394. Fenchel, T. (1980d). The protozoan fauna from the gut of the green turtle, *Chelonia mydas* L. with a description of *Balantidium bacteriophorus* sp. nov. Arch. Protistenk. 123, 22-26.

395. Booyse, D. G., Boomker, E. A., and Dehority, B. A. (2010). Protozoa in the digestive tract of wild herbivores in South Africa. I: Warthogs (*Phacochoerus aethiopicus*). Zootaxa 2492:1, 63-68.

396. Farret, M. H., Fanfa, V. D. R., Silva, A. S. da, and Monteiro, S. G. (2010). Protozoários gastrointestinais em Tayassu pecari mantidos em cativeiro no Brasil. Sem. Ciênc. Agr. 31:4.

397. Neto, J. B., Thatcher, V. E. (1986). Estudos parasitológicos preliminares em tayassuídeos (*Tayassu tajacu*) na Amazônia Central. Rev. Bras. Med. Vet. 8, 175-184.

398. Booyse, D. G., and Dehority, B. A. (2012). Protozoa and digestive tract parameters in Blue wildebeest (*Connochaetes taurinus*) and Black wildebeest (*Connochaetes gnou*), with description of *Entodinium taurinus* n. sp. Europ. J. Protistol. 48:4, 283-289.

399. Vynne, C., and Kinsella, J. M. (2009). First record of entodiniomorph ciliates in a carnivore, the maned wolf (*Chrysocyon brachyurus*), from Brazil. J. Zoo Wildlife Med. 40:2, 382-384.

400. Guirong Su, N. R., Xiang Hua, Z., Zhu, S., and Imai, S. (2000). Rumen Ciliated Protozoan Fauna of the Yak (*Bos grunniens*) in China with the Description of *Entodinium monuo* n. sp. J. Eukaryot. Microbiol. 47:2, 178-182.

401. Booyse, D. G., and Dehority, B. A. (2017). Host-Parasite list updating of Ciliates and fermentation in the digestive tract of wild miscellaneous herbivores in South Africa (RSA). Zootaxa 4258:6, 586-600.

402. Pomajbikova, K., Petrzelkova, K. J., Profousova, I., Modry, D. (2010) Discrepancies in the Occurrence of *Balantidium coli* Between Wild and Captive African Great Apes. J Parasitol. 96:6, 1139-44.

403. Pomajbíková, K., Oborník, M., Horák, A., Petrželková, K. J., Grim, J. N., Levecke, B., Todd, A., Mulama, M., Kiyang, J. (2013) Novel Insights into the Genetic Diversity of *Balantidium* and *Balantidium*-like Cyst-forming Ciliates. PLoS Negl. Trop. Dis. 7:3.

404. Chistyakova, L. V., Kostygov, A. Y, Kornilova, O. A., Yurchenko, V. (2014) Reisolation and redescription of *Balantidium duodeni* Stein, 1867 (Litostomatea, Trichostomatia). Parasitol. Res. 113:11, 4207-15.

405. Fernandez-Galiano D., Campos, M. I. (1992) *Ophryoscolex monoacanthus* n. sp., an Entodiniomorphid Ciliate in the Rumen of the Chamois *Rupricapra rupricapra*. Trans. Am. Microsc. Soc. 111:1, 44-9.

406. Kamler, J. (1999) Infusorial concentration in rumen fluid of red deer, fallow deer, roe deer and moufflon. Acta Vet. Brno. 68:4, 247-52.

407. Crha, J. (1972) Rumen ciliates in Fallow Deer (*Dama dama* L.) in Namest preserve. Acta Vet. Brno. 41:355, 62.
