# Supplementary material for: Trichostomatid Ciliates (Alveolata, Ciliophora, Trichostomatia) Systematics and Diversity: Past, Present, and Future
Source: Front Microbiol. 2020 Jan 15;10:2967. doi: 10.3389/fmicb.2019.02967 (PMC6974537; doi:10.3389/fmicb.2019.02967)
Supplement: MATERIAL S1 — Trichostomatid families and genera. [file Data_Sheet_1.pdf]

Subclass Trichostomatia Bütschli, 1889*

*Diagnoses according to Lynn (2008)

Size, small to large; shape, ovoid to elongate, sometimes with unusual processes and cell appendages; free-swimming; alveoli, typically well-developed, often filled with "skeletal" material; somatic ciliature, variable, from holotrichous to reduced to girdles, bands, or tufts, but with somatic kinetids as for the class; concrement vacuole present in a few forms; extrusomes as somatic mucocysts and oral toxicysts absent; oral region or cavity, typically a densely ciliated vestibulum, with oral cilia sometimes as polybrachykinetids; cytostome, and therefore vestibulum, sometimes antapical; stomatogenesis, telokinetal, but cryptotelokinetal in entodiniomorphids; macronucleus, typically elongate ovoid; micronucleus, present; contractile vacuole, present; cytoproct, often conspicuous; hydrogenosomes, typically replace mitochondria; feeding on bacteria, detritus, plant material ingested by the host, and other ciliates; majority of species endosymbionts in vertebrate hosts; three orders.

Order Vestibuliferida de Puytorac et al., 1974

Somatic ciliature, holotrichous and dense; cortex, often with thick microfilamentous layer between ecto- and endoplasm; oral region a depression or vestibulum, densely ciliated by extensions of somatic kineties, whose cilia do not appear organized as polybrackykinetids; cytostome at base of vestibular cavity; endocommensals in herbivorous placental mammals, but not in marsupials; six families.

Family Balantidiidae Reichenow in Doflein & Reichenow, 1929

Size, small to medium; shape, ovoid to elongate; free-swimming; somatic ciliature, holotrichous, with clavate cilia in several kineties, as possible homologues of the haptorian brosse; no concrement vacuole; extrusomes as somatic mucocysts; oral cavity apico-ventral, as a vestibular groove less than one-half body length and lined by extensions of somatic kineties, which are accompanied by supernumerary kineties; macronucleus, elongate ellipsoid; micronucleus, present; contractile vacuole, present; cytoproct, likely absent; encystment probable; feeding on bacteria and organic detritus, but also may feed on host intestinal epithelial cells; in marine, freshwater, and terrestrial habitats as endosymbionts in the digestive tracts of diverse vertebrate and invertebrate hosts; three genera.

Balantidium Claparede & Lachmann, 1858
Dilleria Earl, 1973
Metacollinia Jankowski, 1980
Balantoides Morey, 1935
Balantidiopsis Bütschli, 1889

Family Isotrichidae Butschli, 1889

Size, small to medium; shape, ovoid, flattened; free-swimming; somatic ciliature, holotrichous, dense, with sometimes up to 200 kineties; no concrement vacuole, but endoplasmic polysaccharide reserves; extrusomes as somatic mucocysts; oral cavity at or near antapical pole, lined by extensions of somatic kineties, with parental vestibulum migrating anteriorly during stomatogenesis to become the vestibulum of the proter; macronucleus, ellipsoid, may be anchored by a karyophore; micronucleus, present; contractile vacuoles, present, may be multiple; cytoproct in posterior; feeding on bacteria and organic detritus; in terrestrial habitats, widely found as endocommensals in ungulate ruminants, but *Protoisotricha* is from rodents, an *Isotricha* was once reported from the cockroach, and Aviisotricha from birds; five genera.

Aviisotricha Bardele et al., 2017
Dasytricha Schuberg, 1888
Isotricha Stein, 1859
Oligoisotricha Imai, 1981
Protoisotricha Kopperi, 1937

Family Paraisotrichidae da Cunha, 1917

Size, small to medium; shape, ovoid to pyriform; free-swimming; somatic ciliature, holotrichous, dense, sometimes slightly spiralled, often with anterior tuft of cilia but ciliature of *Latteuria* confined more to posterior half; extrusomes as somatic mucocysts; concrement vacuole at apical pole; oral region, anterior, with vestibulum ciliated on ventral wall and with cytostome at its base; macronucleus, ellipsoid; micronucleus, present; contractile vacuole, present; feeding on bacteria and organic detritus; in terrestrial habitats, common as endosymbionts in horses , but also in capybaras, guinea pigs, and elephants; three genera and one genus *incertae sedis*.

Latteuria Timoshenko & Imai, 1997
Paraisotricha Fiorentini, 1890
Rhizotricha Wolska, 1964

Incertae sedis in Family Paraisotrichidae
Helicozoster Latteur, 1967

Family Protocaviellidae Grain in Corliss, 1979

Size, small to medium; shape, ovoid to elongate; free-swimming; somatic ciliature, from sparse, somewhat holotrichous to dense holotrichous, sometimes kineties slightly spiralling, with longer cilia as a tuft or band anterior to the oral region; no concrement vacuole; oral region, subapical to subequatorial; oral cavity, funnel- or

trumpet-shaped with adoral polybrachykinety running along the anterior and right edge of the oral cavity opening and vestibular polybrachykinety running along the left wall of the oral cavity, but adoral ciliature absent in *Enterophrya* and *Ogimotopsis*; macronucleus, globular to elongate ellipsoid; micronucleus, present; contractile vacuole, present; cytoproct, present; feeding on bacteria; in terrestrial habitats as endosymbionts in the hindgut of capybaras, guinea pigs, and lagomorphs; nine genera.

Anacharon Ito & Imai, 2000
Cunhamunizia Ito & Imai, 2000
Enterophrya Hasselmann, 1918
Hydrochoerella da Cunha & Muniz, 1925
Ogimotoa Ito & Imai, 2000
Ogimotopsis Ito & Imai, 2000
Paracunhamunizia Ito & Imai, 2000
Protocaviella Kopperi, 1937
Uropogon Ito & Imai, 2000

Family Protohalliidae da Cunha & Muniz, 1927

Size, small; shape, ovoid, with apical disc and posterior anal papilla; free swimming; somatic ciliature, holotrichous; oral region, apical, with oral ciliature short, inconspicuous; adoral ciliature divided into three regions, one densely ciliated, crown-shaped on the left anterior of the oral region and the other two, inconspicuous, on the posterior of the oral region; cytostome apical, with cytopharynx supported by prominent, basket-like cytopharyngeal apparatus; macronucleus, ellipsoid; micronucleus, present; contractile vacuole, present; cytoproct, in posterior; feeding on bacteria, smaller protists, and organic detritus; in terrestrial habitats as endosymbionts in the cecum of capybaras; one genus.

Protohallia da Cunha & Muniz, 1927

Family Pycnotrichidae Poche, 1913

Size, small to large; shape, ovoid; free-swimming; somatic ciliature, holotrichous, dense; cortex, thick, apparently of up to three layers; no concrement vacuole; oral cavity as a long vestibular groove at least one-half body length, lined on its edges by extensions of somatic kineties; cytostome can be subequatorial, posterior or dorsal, depending on the length and orientation of the vestibulum; macronucleus, globular to ellipsoid; micronucleus, present; contractile vacuole, present, sometimes with collecting canals; cytoproct, present; in terrestrial habitats as endosymbionts in various herbivorous vertebrates, including fishes, gundis, hyraxes, capybaras, camels, cattle, water buffaloes, and the red spider monkey; seven genera and one genus *incertae sedis*.

Collinina Chatton & Perard, 1924
Infundibulorium Bozhenko, 1925
Muniziella da Fonseca, 1939
Nicollella Chatton & Perard, 1919
Pycnothrix Schubotz, 1909
Taliaferria Hegner & Rees, 1933
Vestibulongum Grim, 1988

Incertae sedis in Family Pycnotrichidae
Buxtonella Jameson, 1926

Incertae sedis in the Order Vestibuliferida
Microcetella Aescht, 2001

Order Entodiniomorphida Reichenow in Doflein & Reichenow, 1929

Size, small to large; shape, ovoid, often flattened; pellicle firm and thickened, often drawn out into posterior spines; cortex with thick microfilamentous layer between ecto- and endoplasm; somatic ciliature, typically greatly reduced, appearing only in bands, zones or tufts, often as polybrachykineties; concrement vacuole may be present; oral area as only a slight depression to a deep one, often with well-differentiated polybrachykinetids; cytoproct distinct, sometimes at the base of a ciliated tube; in terrestrial habitats, widely found as endosymbionts in mammalian hosts, mainly artiodactyls and perissodactyls, with species of the Family Troglodytellidae restricted to anthropoid apes; three suborders.

Suborder Archistomatina de Puytorac et al., 1974

Size, often small; shape, ovoid to pyriform; free-swimming; somatic ciliature, holotrichous or limited to girdles, tufts or bands; extrusomes as somatic mucocysts; concrement vacuole present, overlain by 4–5 somatic kineties; oral region, apical, with permanent cytostome surrounded by circumoral monokinetids, closely packed and derived from oralized somatic kinetids, as an adoral polybrachykinety; macronucleus, ellipsoid to elongate ellipsoid; micronucleus, present; contractile vacuole, present, sometimes multiple; cytoproct in posterior body portion; in terrestrial habitats as endosymbionts of vertebrates, typically in horses and camels, but also in rodents, hippopotami, and sometimes ruminants; one family.

Family Buetschliidae Poche, 1913

With characteristics of suborder; 32 genera.

Alloiozona Hsiung, 1930
Ampullacula Hsiung, 1930
Amylophorus Pereira & Almeida, 1942

Blepharocodon Bundle, 1895
Blepharoconus Gassovsky, 1919
Blepharomonas Kopperi, 1937
Blepharoplanum Kopperi, 1937
Blepharoposthium Bundle, 1895
Blepharosphaera Bundle, 1895
Blepharozoum Gassovsky, 1919
Buetschlia Schuberg, 1888
Buissonella de Cunha & Muniz, 1925
Bundleia da Cunha & Muniz, 1928
Cucurbella Thurston & Grain, 1971
Didesmis Fiorentini, 1890
Hemiprorodon Strelkow, 1939
Holophryoides Gassovsky, 1919
Holophryozoon Jirovec, 1933
Hsiungella Imai in Aescht, 2001
Kopperia Corliss, 1960
Levanderella Kopperi, 1937
Meiostoma Sandon, 1941
Parabundleia Imai & Ogimoto, 1983
Paraisotrichopsis Gassovsky, 1919
Pingius Hsiung, 1932
Plexobundleia Kornilova, 2005
Polymorphella Corliss, 1960
Prorodonopsis Gassovsky, 1919
Protolutzia da Cunha & Muniz, 1925
Pseudobuetschlia Jirovec, 1933
Sciurula Corliss, 1960
Sulcoarcus Hsiung, 1935
Wolskana Ito, Imai, Ogimoto, & Nakahara, 1996

Suborder Blepharocorythina Wolska, 1971

Size, small; shape, ovoid, laterally flattened, with a prominent frontal lobe in some species, and distinctive corkscrew-like process in others; free swimming; somatic ciliature markedly reduced, as tufts and bands; presumed remnant of concrement vacuole present only as its overlying somatic kinetids; oral area, apical or subapical, with non-retractable cilia; oral cilia inconspicuous, non-retractable, as extensions of somatic ciliature, forming two groups with presumed homologies to those of entodiniomorphines (i.e., the ventral vestibular kineties or vestibular polybrachykinety and the adoral polybrachykinety); macronucleus, globular to ellipsoid; micronucleus, present; contractile vacuole, present; cytoproct, likely in posterior; in terrestrial habitats, as endosymbionts principally in the hindgut of horses, with a few species in elephants, rhinoceros, primates and cattle; one family.

Family Blepharocorythidae Hsiung, 1929

With characteristics of the suborder; nine genera

Blepharocorys Bundle, 1895
Charonina Strand, 1928
Charonnautes Strelkow, 1939
Circodinium Wolska, 1971
Gorilloflasca Ito et al., 2017
Ochoterenaia Chavarria, 1933
Pararaabena Wolska, 1968
Raabena Wolska, 1967
Spirocorys Wolska, 1969

Suborder Entodiniomorphina Reichenow in Doflein & Reichenow, 1929

Size, small to medium, rarely large; shape, typically laterally flattened; somatic ciliature greatly reduced, appearing in tufts, sometimes elongated as spiraled bands, often arranged as polybrachykineties; pellicle firm and thickened, often drawn out into spines; prominent skeletal plates characteristic of many species, composed of polysaccharide reserves, such as - amylopectin granules or plates; oral area, apical to subapical, often retractable; oral cilia organizaed as polybrachykineties, of two parts, a prevestibular band in the peristomial region and a vestibular part(s) sensu stricto; cytoproct distinct; in terrestrial habitats, widely found as endosymbionts in mammal hosts, mainly in artiodactyls and perissodactyls, with species of the Family Troglodytellidae in anthropoid apes; ten families.

Family Cycloposthiidae Poche, 1913

Size, small to large; shape, elongated, often ovoid, with several genera having ununsual finger-like projections (e.g., *Arachnodiniella*, *Phalodinium*); free swimming; somatic cilia, essentially non-retractable, with from none to four caudal tufts or caudalia; skeletal plates, at least one large one, and up to four; oral ciliature in adoral zone, retractable, with adoral polybrachykinety and dorsal perivestibular polybrachykinety; macronucleus, ellipsoid to elongate ellipsoid; micronucleus, present; contractile vacuole, one to several; cytoproct, present; feeding on bacteria and plant fibers; in terrestrial habitats as endosymbionts in the cecum and colon of horses, zebras, rhinoceroses, and tapirs, and occasionally in elephants, capybaras, and hippopotami; 17 genera.

Arachnodinella van Hoven, Gilchrist & Hamilton-Attwell in Aescht, 2001
Bertolinella Carpano, 1941
Bozasella Buisson, 1923
Carinoposthium Jankowski, 1980
Cycloposthium Bundle, 1895

Dicycloposthium Strelkow, 1939
Lavierella Buisson, 1923
Monoposthium Thurston & Noirot-Timothee, 1973
Paracycloposthium Grain, 1994
Phalodinium van Hoven, Gilchrist, & Hamilton-Attwell, 1987
Prototapirella da Cunha, 1918
Rhabdothoracella Aescht, 2001
Toxodinium da Cunha, 1938
Tricaudalia Buisson, 1923
Trifascicularia Strelkow, 1931
Tripalmaria Gassovsky, 1919
Triplumaria Hoare, 1937

Family Gilchristinidae (Ito, Van Hoven, Miyazaki, & Imai, 2006)

Size, medium to large; shape, ellipsoid, laterally flattened; free-swimming; somatic cilia, non-retractable, as several equatorial and/or posterior bands; skeletal plates, one or two; oral ciliature of retractable adoral polybrachykinety, accompanied by paralabial kineties, and with vestibular polybrachykinety extending longitudinally into the oral cavity; macronucleus, elongate; micronucleus, present; contractile vacuoles, dorsal, multiple; cytoproct in posterior body portion; in colon of rhinoceros; two genera.

Digilchristia Ito, Van Hoven, Miyazaki, & Imai, 2006
Gilchristina (Ito, Van Hoven, Miyazaki, & Imai, 2006)

Family Ophryoscolecidae Stein, 1859

Size, small to large; shape, ovoid to fusiform, more or less flattened; free swimming; retractable dorsal ciliary tuft, absent in *Entodinium*, may cover at least 1/3 of body perimeter; skeletal plates commonly present; oral ciliature of retractable adoral polybrachykinety, accompanied by paralabial kineties, with vestibular polybrachykinety extending longitudinally into the oral cavity; macronucleus, typically elongate; micronucleus, present; contractile vacuole, present, variable in number; cytoproct, present; feeding on bacteria, plant detritus, and other ciliates; in terrestrial habitats as endosymbionts in the rumen of artiodactylan ruminants, such as cattle, sheep, goats, deer, antelope, caribou, bison, buffalo, ox, and close relatives, and camels, and intestine of the guinea pig and capybara; 20 genera.

Caloscolex Dogiel, 1926
Campylodinium Jankowski, 1975
Cunhaia Hasselmann, 1924
Diplodinium Schuberg, 1888
Diploplastron Kofoid & MacLennan, 1932
Elytroplastron Kofoid & MacLennan, 1932
Endoralium Eloff & van Hoven, 1980

Enoploplastron Kofoid & MacLennan, 1932
Entodinium Stein, 1859
Eodinium Kofoid & MacLennan, 1932
Epidinium Crawley, 1923
Epiplastron Kofoid & MacLennan, 1933
Eremoplastron Kofoid & MacLennan, 1932
Eudiplodinium Dogiel, 1927
Metadinium Awerinzew & Mutafowa, 1914
Ophryoscolex Stein, 1859
Opisthotrichum Buisson, 1923
Ostracodinium Dogiel, 1927
Polyplastron Dogiel, 1927

Family Parentodiniidae Ito, Miyazaki & Imai, 2002

Size, small; shape, ovoid, laterally compressed, with longitudinal surface striations; free-swimming; somatic ciliature, non-retractable, and no caudalia; no skeleton; oral ciliature as a retractable adoral polybrachykinety, completely encircling vestibular opening, with several vestibular kineties and a set of paralabial kineties to the right of the adoral polybrachykinety; macronucleus, globular to ellipsoid; micronucleus, present; contractile vacuole, present; cytoproct, with ciliary tuft; in terrestrial habitats as endosymbionts in the stomach of hippopotamus and the rumen of cattle; one genus.

Parentodinium Thurston & Noirot-Timothee, 1973

Family Polydiniellidae Corliss, 1960

Size, large; shape, fusiform, slightly laterally flattened; free-swimming; somatic ciliature, non-retractable, as 4–12 “accessory ribbons” partially encircling body; skeletal plates present, variable in number and size; oral ciliature as an adoral zone of non-retractable ciliary tufts; vacuole with granular contents in a caudal lobe that bears cilia; macronucleus, ellipsoid to elongate and twisted; micronucleus, present; contractile vacuoles, small, very numerous, arranged in transverse rows cytoproct, present; feeding on bacteria, flagellates, plant debris, and other organic particles; in terrestrial habitats as endosymbionts in the cecum and colon of elephants only; four genera.

Elephantophilus Kofoid, 1935
Polydiniella Corliss, 1960
Pterodiniella Aescht, 2001
Thoracodinium Latteur, 1958

Family Pseudoentodiniidae Wolska, 1986

Size, small; shape, ovoid, slightly flattened; free-swimming; skeletal plates, anterior, slat-like; somatic ciliature, absent; oral ciliature, a retractable adoral zone of a single, broad adoral polybrachykinety with dorsal part divided into an anterior and posterior fragment and longitudinal files extending along the vestibular wall; macronucleus, ellipsoid; micronucleus, present; contractile vacuole, present; cytoproct, present; in terrestrial habitats as endosymbionts in the digestive tract of elephants; one genus.

Pseudoentodinium Wolska, 1986

Family Rhinozetidae Van Hoven, Gilchrist, & Hamilton-Attwell, 1988

Size, small; shape, ovoid, flattened; free-swimming; somatic ciliary tufts, non-retractable, occurring in three to five short bands on left and right body surfaces; skeletal plates in varying numbers and sizes; oral ciliature as an adoral zone on a retractable cone; macronucleus, elongate band-form; micronucleus, present; contractile vacuoles, 2–6, between macronucleus and cell surface; cytoproct, present; in terrestrial habitats as endosymbionts in the cecum and colon of rhinoceros; one genus.

Rhinozeta van Hoven, Gilchrist, & Hamilton-Attwell, 1988

Family Spirodiniidae Strelkow, 1939

Size, medium; shape, elongated to globose, often markedly laterally flattened; free-swimming; somatic cilia as 2–4 non-retractable ribbons or bands, spiralling around body at different levels; no skeletal plates; oral ciliature, non-retractable, in two bands, with adoral polybrachykinety and dorsal perivestibular polybrachykinety; macronucleus, elongate and band-form; micronucleus, present; contractile vacuole, one or two; cytoproct, present; in terrestrial habitats as endosymbionts predominantly in the colon and cecum of horses; six genera.

Cochliatoxum Gassovsky, 1919
Ditoxum Gassovsky, 1919
Gassovskiella Grain, 1994
Spirodinium Fiorentini, 1890
Tetratoxum Gassovsky, 1919
Triadinium Fiorentini, 1890

Family Telamodiniidae Latteur & Dufey, 1967

Size, medium; shape, elongate; free-swimming; somatic cilia as five, non-retractable, “accessory ciliary ribbons” partially encircling body; two or three skeletal plates present; oral ciliature of adoral zone, retractable; macronucleus, elongate; micronucleus, present; contractile vacuole, present, may be multiple;

cytoproct, present; in terrestrial habitats as endosymbionts in the colon of the desert wart-hog; three genera.

Megadinium Latteur & Dufey, 1967
Telamodinium Latteur & Dufey, 1967
Teratodinium Latteur & Dufey, 1967

Family Troglodytellidae Corliss, 1979

Size, medium to large; shape, ovoid, fusiform, laterally flattened; free-swimming; somatic cilia as 3–5 non-retractable bands encircling body, essentially perpendicular to longitudinal axis; skeletal plates, large, both dorsal and ventral, fusing to envelope anterior half of organism; cell surface between ciliary bands divided by deep cortical grooves into elongated rectangles; oral ciliature of adoral zone, retractable, polybrachykinety; macronucleus, L-shaped; micronucleus, present; contractile vacuole, multiple, in transverse rows; cytoproct, present; feeding on bacteria and detritus; in terrestrial habitats as endosymbionts in the colon of anthropoid apes only; two genera.

Gorillophilus Imai, Ikeda, Collet, & Bonhomme, 1991
Troglodytella Brumpt & Joyeux, 1912

Order Macropodiniida

Size, small to medium; shape, ovoid to elongate; free-swimming; somatic ciliature, holotrichous, but may be reduced to a dorso ventral groove in macropodiniids; extrusomes as somatic mucocysts; oral cavity, anterior, a shallow to deep vestibulum lined by extensions of somatic kineties, and supported by nematodesmata arising from these kinetids; stomatogenesis, telokinetal or cryptotelokinetal, possibly apokinetal; macronucleus, spheroid to elongate band-form; micronucleus, present; contractile vacuole, present; cytoproct, present; feeding on bacteria, starch, and in larger forms, other ciliates; in terrestrial habitats as endosymbionts in the forestomach of macropodid and vombatid marsupials; three families.

Family Amylovoracidae Cameron & O'Donoghue, 2002

Size, small to medium; shape, ovoid, slightly flattened; free-swimming; somatic ciliature, holotrichous, sometimes spiralling and/or separated into longitudinal bands of closely adjacent kineties by broad interkinetal ridges; extrusomes as somatic mucocysts; oral ciliature as extensions of somatic kineties or in isolated vestibular fields; macronucleus, ellipsoid to elongate band form; micronucleus, present; contractile vacuole, present; cytoproct, may be present; feeding mainly on starch and bacteria; in terrestrial habitats as endosymbionts in the forestomach of macropodid and vombatid marsupials; three genera.

Amylovorax Cameron & O'Donoghue, 2002
Bandia Cameron & O'Donoghue, 2002
Bitricha Cameron, O'Donoghue, & Adlard,2000

Family Macropodiniidae Dehority, 1996

Size, small to medium; shape, ovoid, flattened; free-swimming; cortical alveoli divided by transverse grooves, elaborated into strikingly trapezoidal and parallelogram shapes, filled with dense material; somatic cilia, holotrichous or restricted to kineties lying in a dorso ventral groove that encircles the body; extrusomes as somatic mucocysts; oral cavity, conical, bordered or lined by extensions of somatic kineties; macronucleus, globular to ellipsoid; micronucleus, present; contractile vacuole, present; cytoproct, present; feeding on bacteria, starch grains, and smaller ciliates; in terrestrial habitats as endosymbionts in the stomach of macropodid marsupials; one genus and one genus *incertae sedis*.

Macropodinium Dehority, 1996

Incertae sedis in Family Macropodiniidae
Megavestibulum Cameron & O'Donoghue, 2003

Family Polycostidae Cameron & O'Donoghue, 2003

Size, small to medium; shape, stout ovoid; free-swimming; somatic ciliature, holotrichous, with meridional kineties that can be separated by broad interkinetal ridges filled with many, small dense bodies; oral cavity, a conical, round or flattened vestibulum in cross section, lined by extensions of some somatic kineties only along right side; macronucleus, globular to ellipsoid; micronucleus, present; contractile vacuole, one or more; cytoproct, present; feeding on bacteria and starch; in terrestrial habitats as endosymbionts in the forestomach of macropodid marsupials; one genus.

Polycosta Cameron & O'Donoghue, 2003
