# Supplementary material for: Trichostomatid Ciliates (Alveolata, Ciliophora, Trichostomatia) Systematics and Diversity: Past, Present, and Future
Source: Front Microbiol. 2020 Jan 15;10:2967. doi: 10.3389/fmicb.2019.02967 (PMC6974537; doi:10.3389/fmicb.2019.02967)
Supplement: MATERIAL S2 — History of classification of subclass Trichostomatia. [file Data_Sheet_2.pdf]

Stein 1858-1858
Infusoria<br>Enterodela<br>Family Ophryocercina
**Bütschli 1887-1889**
Class Infusoria<br>Subclass Ciliata<br>1. Order Holotricha<br>Suborder Trichostomata<br>Family Isotrichidae<br>2. Order Heterotricha<br>Suborder Oligotricha<br>Family Ophryoscolecidae
**Kahlian Era 1930-1950**
Subphylum Ciliophora<br>Class Ciliata<br>Subclass Euciliata<br>1. Order Holotricha<br>Suborder Gymnostomata<br>Family Buetschliidae<br>Suborder Trichostomata<br>Family Balantidiidae<br>Family Cyathodiniidae<br>Family Isotrichidae<br>Family Paraisotrichidae<br>Family Protohaliidae<br>Family Pycnotrichidae<br>Family Blepharocorythidae<br>2. Order Entodiniomorpha<br>Family Cycloposthiidae<br>Family Ditoxidae<br>Family Ophryoscolecidae<br>Family Spirodiniidae
**Faurean Era 1950-1970**
Subphylum Ciliophora<br>Class Ciliata<br>Subclass Holotricha<br>1. Order Gymnostomata<br>Family Buetschliidae<br>2. Order Trichostomatida<br>Family Balantidiidae<br>Family Cyathodiniidae<br>Family Isotrichidae

Family Paraisotrichidae
Family Protohaliidae
Family Pycnotrichidae
Family Blepharocorythidae

3. Order Spirotricha

Suborder Entodiniomorpha
Family Cycloposthiidae
Family Ditoxidae
Family Ophryoscolecidae
Family Polydiniellidae
Family Spirodiniidae
Family Telamodiniidae

Post Faurean Era 1970-1981

Phylum Ciliophora
Class Kinetofragninophora
Subclass Gymnostomata
Family Buetschliidae
Subclass Vestibulifera

1. Order Trichostomatida

Suborder Trichostomatina
Family Balantidiidae
Family Cyathodiniidae
Family Paraisotrichidae
Family Protohaliidae
Family Protocaviellidae
Family Pycnotrichidae
Suborder Blepharocorythina
Family Blepharocorythidae

2. Order Entodiniomorpha

Family Cycloposthiidae
Family Ditoxidae
Family Ophryoscolecidae
Family Spirodiniidae
Family Telamodiniidae
Family Troglodyteliidae

Small & Lynn 1981

Phylum Ciliophora
Subphylum Postciliodermatophora
Class Litostomea
Subclass Vestibuliferia

1. Order Archistomatina

Family Buetschliidae
Order Trichostomatida
Family Balantidiidae

Family Cyathodiniidae
Family Isotrichidae
Family Paraisotrichidae
Family Protohaliidae
Family Pycnotrichidae
Suborder Entodiniomorphida
Family Cycloposthiidae
Family Ditoxidae
Family Ophryoscolecidae
Family Polydiniellidae
Family Telamodiniidae
Family Troglodytellidae
**Puytorac & Mignot 1987**
Phylum Ciliophora
Subphylum Prostomatea
Subclass Archistomatia
1. Order Archistomatina
Family Buetschliidae
Class Vestibuliferea
2. Order Trichostomatida
Suborder Trichostomatina
Family Balantidiidae
Family Cyathodiniidae
Family Isotrichidae
Family Paraisotrichidae
Family Protohaliidae
Family Pycnotrichidae
Suborder Blepharocorythina
Family Blepharocorythidae
3. Order Entodiniomorphida
Family Cycloposthiidae
Family Ditoxidae
Family Ophryoscolecidae
Family Rhinozetidae
Family Spirodiniidae
Family Polydiniellidae
Family Pseudoentodiniidae
Family Telamodiniidae
Family Troglodytellidae
**Lynn 2008**
Phylum Ciliophora
Subphylum Intramacronucleata
Class Litostomatea
Subclass Trichostomatia
1. Order Vestibuliferida
Family Balantidiidae
Family Isotrichidae

Family Paraisotrichidae
Family Protocaviellidae
Family Protohallidae
Family Pynotrichidae
2. Order Entodiniomorphida
Subrder Archistomatina
Family Buetschliidae
Suborder Blepharocorythina
Family Blepharocorythidae
Suborder Entodiniomorphina
Family Cycloposthiidae
Family Gilchristinidae
Family Ophryoscolecidae
Family Parentodiniidae
Family Polydiniellidae
Family Pseudoentodiniidae
Family Rhinozetidae
Family Spirodiniidae
Family Telamodiniidae
Family Troglodytellidae
3. Order Macropodiniida
Family Amylovoracidae
Family Macropodiniidae
Family Polycostidae
